# The Sirt1‐Piezo1 Axis Promotes Bone Formation and Repair in Mice

**DOI:** 10.1002/advs.202510103

**Published:** 2025-09-26

**Authors:** Donghao Gan, Yi Ran, Hong Pan, Qinnan Yan, Wenjing Zhang, Bo Zhou, Pengzhi Xu, Rongdong Liao, Haipeng Xue, Will Jiang, Tailin He, Qingyun Jia, Lei Qin, Francis Y Lee, Di Chen, Chuanju Liu, Guozhi Xiao

**Affiliations:** ^1^ Department of Biochemistry Homeostatic Medicine Institute School of Medicine Guangdong Provincial Key Laboratory of Cell Microenvironment and Disease Research Shenzhen Key Laboratory of Cell Microenvironment Southern University of Science and Technology Shenzhen 518055 China; ^2^ Guangdong Key Laboratory of Nanomedicine CAS‐HK Joint Lab of Biomaterials Shenzhen Institute of Advanced Technology Chinese Academy of Sciences Shenzhen 518055 China; ^3^ University of Chinese Academy of Sciences Beijing 101408 China; ^4^ School of Medicine Shenzhen University Shenzhen 518055 China; ^5^ Department of Orthopedics Linyi People's Hospital Linyi 276800 China; ^6^ Department of Joint and Orthopedics Zhujiang Hospital Southern Medical University Guangzhou 510282 China; ^7^ Department of Orthopedics Affiliated Hospital of Shandong University of Traditional Chinese Medicine Jinan 250014 China; ^8^ Department of Orthopaedics & Rehabilitation Yale University School of Medicine New Haven 06510 USA; ^9^ Department of Orthopedics Huazhong University of Science and Technology Union Shenzhen Hospital Shenzhen 518000 China; ^10^ Faculty of Pharmaceutical Sciences Shenzhen University of Advanced Technology Shenzhen 518107 China

**Keywords:** bone repair, chondrocytes, deacetylation, osteogenesis, Piezo1, Sirt1

## Abstract

The mechanosensitive Piezo1 channel protein plays a pivotal role in promoting bone formation and repair; however, its underlying molecular mechanism(s) are poorly defined. Here this study shows that Sirt1 positively regulates Piezo1 expression and activity to promote osteogenesis and bone repair in mice. This study finds that Piezo1 is up‐regulated in the cartilage callus during bone repair. Deleting Piezo1 in chondrocytes largely impairs endochondral ossification and mechanically induced osteogenesis and delays fracture healing in mice, while Yoda1 activation of Piezo1 exerts opposite effects. Sirt1 overexpression or activation dramatically increases Piezo1 protein expression in a dose‐dependent manner. Sirt1 binds to Piezo1 protein and deacetylates and activates Piezo1 and Ca^2+^ influx in chondrocytes. Piezo1 loss in chondrocytes abolishes the ability of Sirt1 activator SRT2104 to accelerate bone repair. Resveratrol (RSV), a natural Sirt1 activator, also potently activates Piezo1 and enhances bone repair. A yeast microcapsule‐based oral formulation of RSV (YC‐RSV) is developed to improve drug bioavailability and therapeutic efficacy, which highly and selectively targets to the inflammatory fracture site. Thus, it demonstrates that Sirt1 is a novel and potent activator of Piezo1 to promote bone formation and repair, supporting the potential clinical application of Sirt1 activators in promoting bone formation and repair.

## Graphical abstract

Piezo1 upregulates in cartilage callus during bone repair, where its deletion impairs ossification and healing in mice, while activation enhances these processes. Sirt1 positively regulates Piezo1 expression and activity to promote osteogenesis and bone repair in mice. Resveratrol potently activates Piezo1, and a novel yeast microcapsule formulation targets fractures, advancing Sirt1 activators for clinical bone repair.

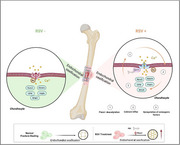

## 1 Introduction

Mechanical sensitive ion channels are molecular force sensors for cells, specifically designed to rapidly convert various mechanical stimuli into biochemical signals to regulate specific cellular and physiological responses. Piezo1 is a large ion channel protein that is widely expressed in different tissues and organs and participates in specific physiological and pathological processes by sensing mechanical stresses.^[^ ^1^, ^2^ ^]^ Piezo1 is sensitive to membrane tension and lipid composition,^[^ ^3^, ^4^ ^]^ and is activated not only by mechanical stimulation, but also by chemicals or protein–protein interactions.^[^ ^5^ ^]^ Compounds such as Jedi1/2 and Yoda1 can activate Piezo1 channel,^[^ ^6^, ^7^ ^]^ while GsMTx4, a spider venom peptide, reversibly inhibits the current of cellular Piezo1.^[^ ^8^ ^]^ In addition, post‐translational modification affects the activity and expression of Piezo family proteins. N‐chain glycosylation of PIEZO1 was reported to cause membrane transport defects in PIEZO1.^[^ ^3^ ^]^ PKA phosphorylation regulates PIEZO2 current induced by focal mechanical indentation of cells.^[^ ^9^ ^]^

Acetylation is a common post‐translational modification that regulates protein activity, stability, crosstalk with other post‐translational modifications, and protein–protein and protein‐DNA interactions.^[^ ^10^ ^]^ Silent information regulator 1 (SIRT1) is a highly conserved nicotinamide adenine dinucleotide (NAD+) ‐dependent deacetylase, which regulates various physiological and pathological processes, such as immune inflammation, oxidative stress, metabolism and aging by deacetylating proteins.^[^ ^11^ ^]^ Deacetylation was reported to regulate ion channel activation. Chiu et al. found that GPCR‐mediated activation of Pannexin 1 can occur through lysine deacetylation.^[^ ^12^ ^]^ SIRT1 rapidly regulated the anxious behavior and synaptic properties of granular cells in the dentate gyrus through deacetylation of the *α* subunit of the BK channel.^[^ ^13^ ^]^ Recent studies report that SIRT1 improves bone mass and prevents fractures in mice,^[^ ^14^ ^]^ but its underlying molecular insight remains poorly defined.

Mechanical environment is known to play a key role in fracture repair and bone formation.^[^ ^15^, ^16^ ^]^ Cumulative evidence reveals that Piezo1 mediates mechanically induced bone formation via actions in osteoblasts and chondrocytes and is essential for skeletal development and repair.^[^ ^17^, ^18^, ^19^ ^]^ However, there is currently very limited knowledge available regarding how Piezo1 activity is modulated under physiological and pathological conditions. Better understanding of this knowledge will help on designing new strategies to promote bone formation and repair.

In this study, we demonstrate that Sirt1 is a novel and a novel modulator of the Piezo1 ion channel by promoting Piezo1 protein expression and activity. By doing so, Sirt1 promotes bone formation and repairs in multiple mouse models. Yeast microcapsules (YCs), derived from edible yeast cell walls, exhibit favorable biocompatibility, macrophage‐targeting and drug‐loading capacity, enabling efficient small‐molecule agonist delivery though macrophage inflammatory chemotaxis in vivo.^[^ ^20^, ^21^ ^]^ Of potential translational significance, we have developed a novel yeast microcapsule‐based delivery system for oral administration and fracture site‐specific delivery for RSV and demonstrate that RSV potently promotes bone formation and fracture repair in vivo.

## 2 Results

### 2.1 Piezo1 Up‐Regulation in Cartilage Callus During Mechanically Induced Osteogenesis and Bone Repair

**Figure 1:**
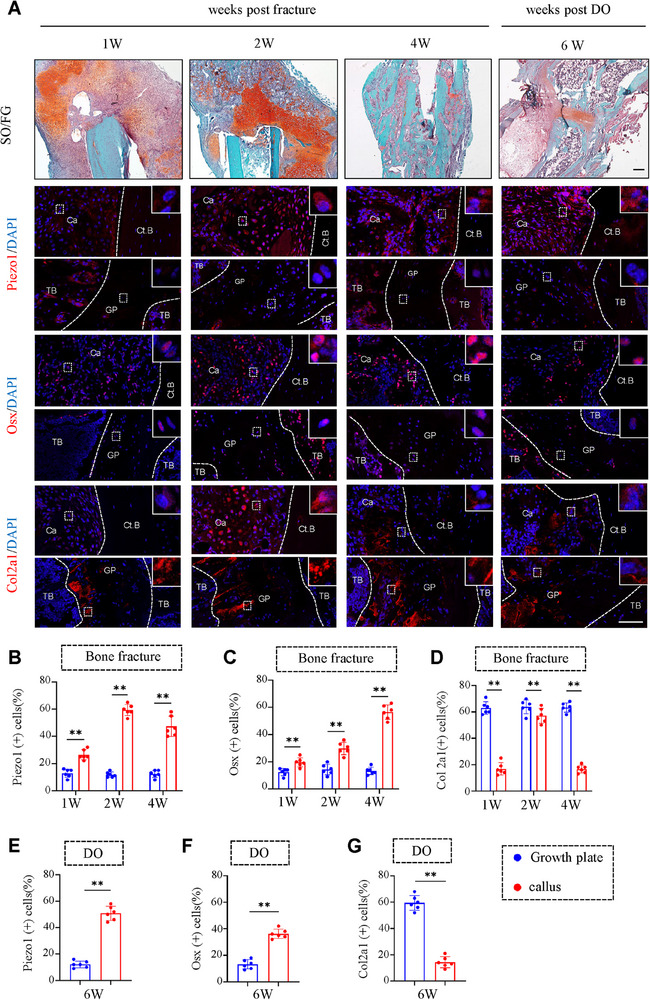
Piezo1 expression in chondrocytes is up‐regulated during fracture repair. A) Representative images of SO/FG and IF staining of Piezo1, osterix (Osx) and Col2a1 in cartilage calluses (upper panel) or growth plate(lower panel) at 1, 2 and 4 weeks post fracture or 6 weeks post DO surgery, with the bone cortex or growth plate inside the dotted white lines. SO/FG staining scale bar: 200 µm; IF staining scale bar: 100 µm. B‐D) Quantification of Piezo1, Osx, and Col2a1 positive cells in calluses based on fracture model staining based on (A). E‐G) Quantification of Piezo1, Osx, and Col2a1 positive cells in calluses based on DO model staining based on (A). N = 6 per group. Results are expressed as mean ± standard deviation (s.d.). **P < 0.01. Ct.B, cortical bone; Ca, callus; TB, trabecula bone; Veh, vehicle; GP, grow plate.

As an initial step to investigate potential role of the mechanosensitive ion channel Piezo1 in regulation of osteogenesis and fracture healing, we determined its expression in the process of fracture healing process. First, we examined the expression changes of Piezo1 during fracture healing. We first constructed a mouse model of intramedullary nail fixation of femoral fracture, and collected samples at 1/2/4 weeks after surgery. Results from IF staining showed that, 1 week after surgery, there was significant expression of Piezo1, Osx and Col2a1 in the cartilage callus area (**Figure** **1**A‐D), and Piezo1 and Osx expression levels were increased during the healing process (Figure 1A‐D). We then established a mouse femur distractive osteogenesis (DO) model and analyzed the samples after 6 weeks of distraction. Results showed that the expression levels of Piezo1 in cartilage callus were higher than those in resting or proliferating chondrocytes in the growth plate, and were positively correlated with osteogenic protein (Figure 1A,E,F). Moreover, six weeks after DO, the expression levels of cartilage marker protein decreased as osteogenic protein levels increased (Figure 1A,E,G). To further investigate the dynamic changes of Piezo1 expression during fracture callus formation, we quantified Piezo1 expression in chondrocytes at single‐cell resolution, we reanalyzed a previously published single‐cell sequencing dataset, GSE150291, which was derived from single‐cell RNA sequencing of non‐hematopoietic cells extracted from the bone marrow of 4‐month‐old mice. We characterized the differentiation of mesenchymal stem/stromal cells (MSCs) with RNA velocity (Figure S1A,B, Supporting Information). The Piezo family members Piezo1 and Piezo2 showed distinct expression patterns in chondrocytes (Figure S1C, Supporting Information). At single cell level, the expression of Piezo1 was highly and specifically enriched in chondrocyte lineage, while that of Piezo2 was absent in chondrocytes (Figure S1D,E, Supporting Information). We next analyzed the single‐cell RNA sequencing dataset GSE154247. In this study, femurs were harvested at post‐fracture day 14 (PFD14) and from uninjured controls. Single‐cell suspensions were prepared from both control and PFD14 bones, and non‐hematopoietic stromal cells were isolated for single‐cell RNA sequencing. As revealed by single cell profiling, the proportion of chondrocytes was dramatically expanded at post fracture day 14, when compared with that in control (Figure S1F‐H, Supporting Information). Furthermore, differential gene expression analysis showed 844 up‐regulated genes and 2489 down‐regulated genes in chondrocytes, including those involved in chondrocyte differentiation and ossification (Figure S1I, Supporting Information). The expression of endochondral ossification‐related genes, including those encoding osterix (Osx), Col1a1, Alp and Vegfa, was up‐regulated in chondrocytes after fracture (Figure S1J‐M, Supporting Information). Piezo1 expression was up‐regulated at post fracture day 14 (Figure S1N, Supporting Information). We confirmed that the expression of PIEZO1 was up‐regulated in human cartilage callus compared to that in the control tissue (Figure S1O,P, Supporting Information).

### 2.2 Deleting Piezo1 in Chondrocyte Impairs Endochondral Ossification, Fracture Healing and Mechanically Induced Bone Formation in Mice

**Figure 2:**
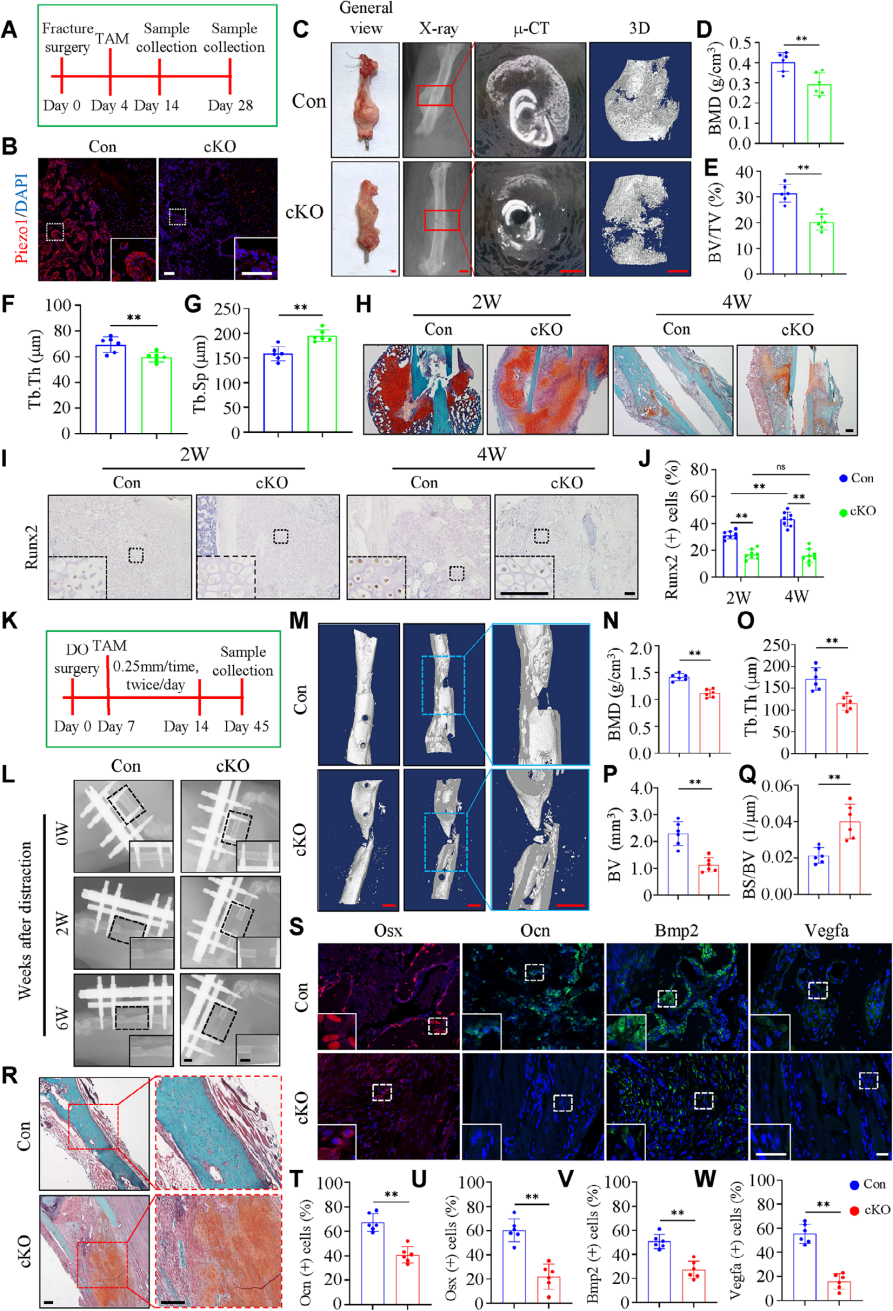
Piezo1 loss in chondrocytes impairs osteogenesis and fracture healing by impairing endochondral ossification. A) Schematic diagram of bone fracture experimental design. B) Representative images of IF staining of Piezo1 in callus of TAM‐induced cKO and control mice. Scale bar: 50 µm. C) General view and 2D and 3D reconstruction from µCT scans of calluses from cKO and control mice at 4 weeks after fracture surgery. Scale bar: 1.0 mm. D‐G) The bone mineral density (BMD) (D), bone volume/tissue volume (BV/TV) (E), trabecular thickness (Tb.Th) (F), and trabecular separation (Tb.Sp) (G) of callus were analyzed based on µCT results in (C). H) Representative images of SO/FG staining of control and cKO callus at 2 and 4 weeks after fracture surgery. Scale bar: 200 µm. I) Representative images of IHC staining of Runx2 in callus cells of both groups at 2 and 4 weeks after fracture surgery. Scale bar: 50 µm. J) Quantification of Runx2‐positive cells based on staining results in (I). K) Schematic diagram illustrating the DO experimental design. L) Representative X‐ray images of callus in control and cKO mice 0/2/6 weeks after DO surgery. Scale bar: 1.0 mm. M) 3D reconstruction from µCT scans of callus from control and cKO mice at 6 weeks after DO surgery. Scale bar: 1.0 mm. N‐Q) The BMD (N), Tb.Th (O), BV (P), and bone surface/bone volume (BS/BV) (Q) of callus were analyzed based on µCT results in (M). R) Representative images of H/E staining of control and cKO callus at 6 weeks after DO surgery. Scale bar: 200 µm. S) Representative images of IF staining of Osx, Ocn, Bmp2 and Vegfa in callus cells of both groups at 6 weeks after DO surgery. Scale bar: 50 µm. T‐W) Quantification of Osx, Ocn, Bmp2 and Vegfa positive cells based on IF staining results in (S). N = 6 per group. Results are expressed as mean ± standard deviation (s.d.). **P < 0.01.

To further investigate whether Piezo1 is involved in fracture healing, we constructed femur fracture models in mice with Piezo1 deletion in chondrocytes. Four days after surgery, TAM (100 mg kg^−1^ body weight) was injected into *Piezo1^flox/flox^; Aggrecan^CreERT2^* mice to induce Cre expression to delete Piezo1 in chondrocytes (cKO) (**Figure** **2**A). Sex‐ and age‐matched *Piezo1^flox/flox^* mice injected with TAM were used as controls in this study. Results showed that Piezo1 expression was drastically reduced in callus chondrocytes of cKO mice, as revealed by IF staining (Figure 2B). X‐ray, µCT and 3D reconstruction analyses were performed on the fracture sites of mice at 4 weeks post fracture. Results showed that the cKO group had significantly smaller callus than that in the control group, (Figure 2C). When compared to control group, the callus in the cKO group was less mineralized and displayed larger unmineralized gaps (Figure 2D‐G). SO/FG staining showed that at 2 and 4 weeks post fracture, the cKO group had fewer bone trabeculae and more cartilage tissue (Figure 2H), as well as the lower proportion of Runx2 positive cells in callus (Figure 2I,J). Collectively, these results demonstrate that Piezo1 loss in chondrocytes impairs callus mineralization and delays fracture healing by primarily impairing the endochondral ossification.

We further investigated the role of Piezo1 in chondrocytes during DO process. We successfully established the DO model in mice. Stretching started 1 week post surgery and lasted for 1 week. TAM injection began 1 day before stretch to induce Cre expression and Piezo1 deletion in callus chondrocytes (Figure 2K). X‐ray analysis of stretch sites in mice was performed 0/2/6 weeks after stretch surgery (Figure 2L). Six weeks after surgery, samples were collected for µCT and 3D reconstruction analyses. cKO group showed reduced bone mass in the distraction gap (Figure 2M‐Q). cKO group had a large amount of fibrous tissue in the distraction gap, and the bone trabeculae did not close the gap (Figure 2R). Piezo1 ablation decreased the proportion of Osx‐, Ocn‐, Bmp2‐ and Vegf‐positive cells in the callus (Figure 2S‐W). Thus, we demonstrate that Piezo1 loss in chondrocytes inhibits mechanically induced osteogenesis in mice.

Piezo1 loss in chondrocytes increased the width of the growth plate (Figure S2A‐E, Supporting Information), inhibited the endochondral ossification of the growth plate (Figure S2H‐K, Supporting Information), and increased the osteoclast formation (Figure S2C,F,G, Supporting Information).

### 2.3 Piezo1 Activation Promotes Osteogenesis and Bone Repair in Mice

**Figure 3:**
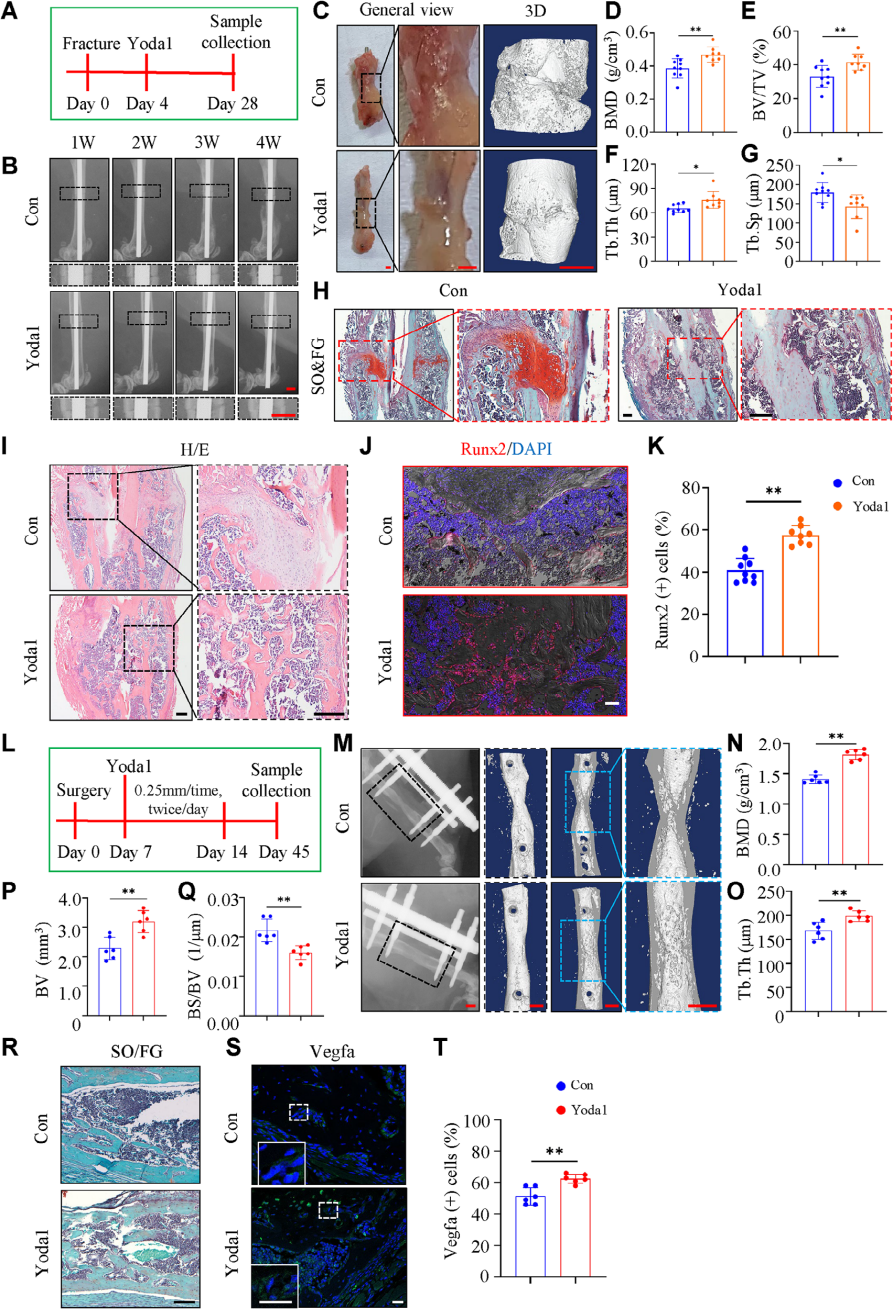
Piezo1 activation promotes bone healing and distraction osteogenesis in mice. A) Schematic diagram illustrating the bone fracture experimental design. B) Representative X‐ray images of mice callus in control and Yoda1 groups at 1/2/3/4 weeks after fracture surgery. Scale bar: 1.0 mm. C) Callus appearance and 3D reconstructions from µCT scans of both group mice at 4 weeks after fracture surgery. Scale bar: 1.0 mm. D‐G) The BMD (D), BV/TV (E), Tb.Th (F), and Tb.Sp (G) of callus were analyzed based on µCT results in (C). H) Representative images of SO/FG staining of both group mice callus at 4 weeks after fracture surgery. Scale bar: 200 µm. I) Representative images of H/E staining of both group mice callus at 4 weeks after fracture surgery. Scale bar: 200 µm. J) Representative images of IF staining of Runx2 in callus cells of both groups at 4 weeks after fracture surgery. Scale bar: 50 µm. K) Quantification of Runx2 positive cells based on IF staining results in (J). L) Schematic diagram illustrating the DO experimental design. M) Representative X‐ray and 3D reconstruction images from µCT scans of callus from control and Yoda1 mice at 6 weeks after DO surgery. Scale bar: 1.0 mm. N‐Q) The BMD (N), Tb.Th (O), BV (P), and BS/BV (Q) of callus were analyzed based on µCT results in (M). R) Representative images of SO/FG staining of both group callus at 6 weeks after DO surgery. Scale bar: 200 µm. S) Representative images of IF staining of Vegfa in callus cells of both groups at 6 weeks after DO surgery. Scale bar: 50 µm. T) Quantification of Vegfa positive cells based on IF staining results in (S). N = 6 per group. Results are expressed as mean ± standard deviation (s.d.). **P < 0.01.

We next investigated the effect of Piezo1 activation in the callus on fracture healing. Yoda1 is a selective activator of Piezo1. We first utilized the femoral fracture model in 12‐week‐old C57BL/6 male mice. Yoda1 or equal volume of solvent was injected into the fracture gap, and samples were collected for analysis 4 weeks post fracture (**Figure** **3**A). Results revealed that Yoda1 treatment promoted fracture healing by enhancing callus mineralization and increasing the trabecular bone volume and mineral density (Figure 3B‐I). Yoda1 increased expression of osteogenic proteins, such as Runx2 (Figure 3J,K). We also investigated the effect of Piezo1 activation on osteogenesis in the mouse DO model. Yoda1 or equal volume of solvent was applied to the distraction gap in 12‐week‐old C57BL/6 male mice, and samples were collected for analysis 45 days post operation (Figure 3L). Results showed that Yoda1 treatment increased callus mineralization, thickened bone trabeculae, accelerated closing of the fracture gap (Figure 3M‐R), and increased the expression of vascular formation related proteins (Figure 3S,T)

### 2.4 Sirt1 Promotes Piezo1 Protein Expression and Activity

**Figure 4:**
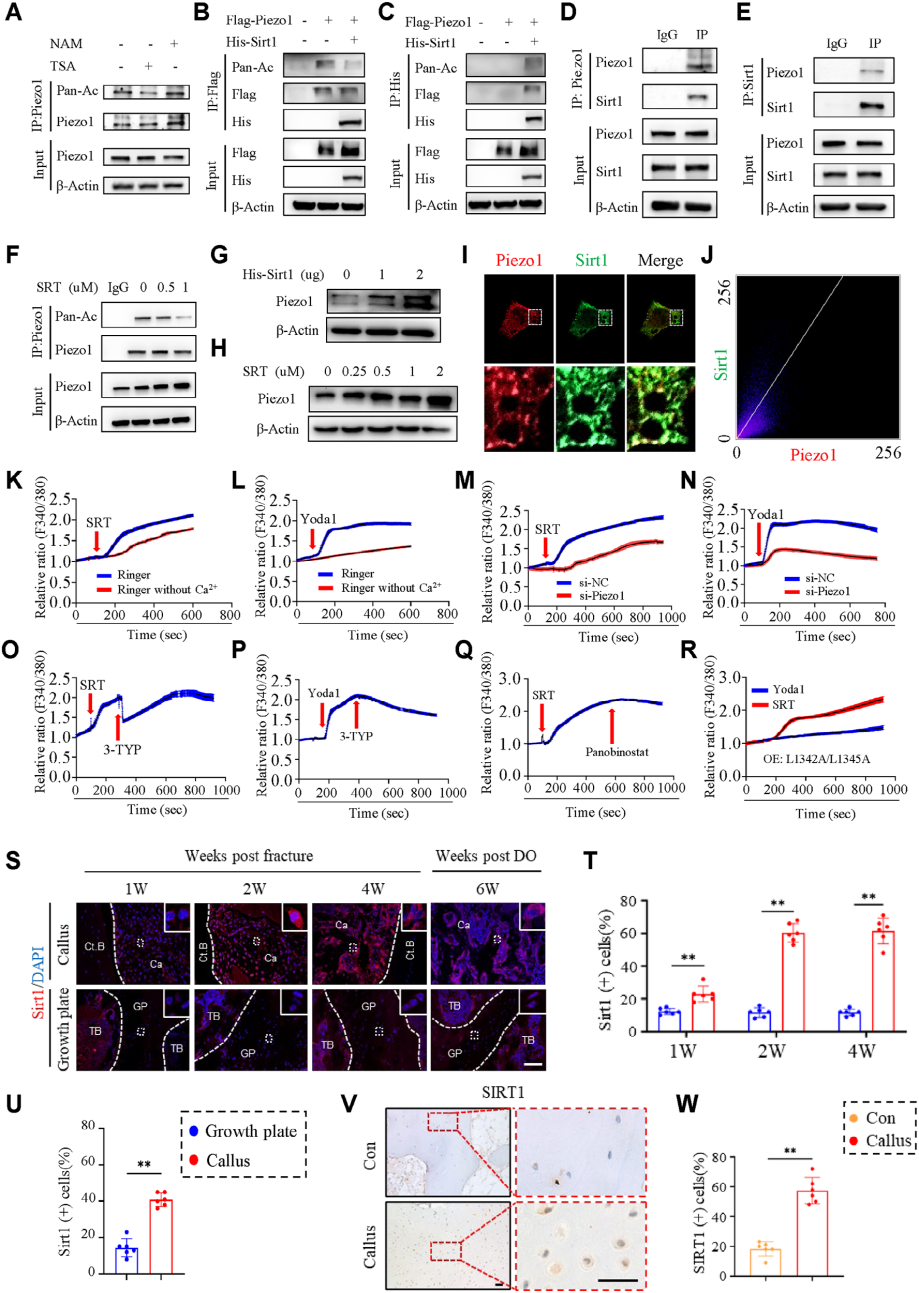
Sirt1 binds to, deacetylates and activates Piezo1. A) ATDC5 cells were treated with 10 µM Trichostatin A (TSA) and 5 µM Nicotinamide (NAM) for 24 h, followed by immunoprecipitation (IP) and western blotting (IB) with the indicated antibodies. B) Transient transfection of HEK293T cells with Flag‐Piezo1 and His‐Sirt1 plasmids, followed by IP and IB with the indicated antibodies 48 h after transfection. C) Transient transfection of HEK293T cells with Flag‐Piezo1 and His‐Sirt1 plasmids, followed by IP and IB with the indicated antibodies 48 h after transfection. D) Whole cell extracts were prepared from ATDC5 cells, followed by IP and IB with the indicated antibodies. E) Whole cell extracts were prepared from ATDC5 cells, followed by IP and IB with the indicated antibodies. F) ATDC5 cells were treated with different concentrations of SRT2104 (SRT), a specific Sirt1 activator, for 48 h, followed by IP and IB using the indicated antibodies. G) After transfection with His‐Sirt1 plasmid into HEK293T cells for 48 h, western blotting was used to detect the expression of Piezo1 protein in the cells. H) After SRT intervention in ATDC5 cells for 48 h, western blotting was used to detect the expression of Piezo1 protein in the cells. I) Representative confocal images showing colocalization of Piezo1 (red) and Sirt1 (green) in ATDC5 cells. A zoomed view of the insets is shown on the second side of the colour panels. J) Scatter plot of colocalization analysis of Piezo1 and Sirt1 with Colocalization‐Coloc 2. K,L) Representative Fura‐2 ratio (340/380) traces obtained from single‐cell Ca^2+^ imaging of ATDC5 in response to the indicated conditions. M,N) Representative Fura‐2 ratio (340/380) traces obtained from single‐cell Ca^2+^ imaging of ATDC5 cells treated with SRT (5 µM) or Yoda1 (1 µM) after transfected with si‐NC or si‐Piezo1 for 48 h, respectively. O,P) Single‐cell Fura‐2 Ca^2+^ imaging experiments showing the average response of ATDC5 cells treated with SRT or Yoda1 and 3‐TYP (1 µM). Q) Single‐cell Fura‐2 Ca^2+^ imaging experiments showing the average response of ATDC5 cells treated with SRT and panobinostat (10 nM). R) Representative Fura‐2 ratio (340/380) traces obtained from single‐cell Ca^2+^ imaging of HEK293T cells treated with Yoda1 or SRT after transfected with L1341A/L1344A mutant plasmid, respectively. The black trace represents the average response of 3‐dish repeats. Error bars are shown in colors. Note: All chemicals at experimental concentration did not impact the cell viability. S) Representative images of IF staining of Sirt1 in calluses and growth plate at 1, 2 and 4 weeks post fracture or 6 weeks post DO surgery, with the bone cortex inside the dotted white lines. Scale bar: 100 µm. T) Quantification of Sirt1 positive cells in calluses and growth plate based on fracture model staining based on(S). U) Quantification of Sirt1 positive cells in calluses and growth plate based on DO model staining based on (S). V) Representative images of IHC staining of SIRT1 in human cartilage calluses or cortical bone. Scale bar: 50 µm. W) Quantification of SIRT1 positive cells in calluses or cortical bone based on (V). N = 6 per group. Results are expressed as mean ± standard deviation (s.d.). **P < 0.01. Ct.B = cortical bone; Ca = callus; GP = growth plate.

In our effort to explore post‐translational modification regulation of Piezo1 activation, we firstly predicted the presence of potential acetylation sites in Piezo1 molecule through Deep‐PLA website (http://deeppla.omicsbio.info/) and then studied the effects of the deacetylation of Piezo1 on its activity. The acetylation level of Piezo1 was elevated by inhibiting the activity of deacetylase, indicating the presence of acetylation modification in Piezo1 (**Figure** **4**A). Next, we investigated the effect of the deacetylation activator Sirt1 on Piezo1 acetylation. We found that Sirt1 can interact with Piezo1 either directly or indirectly, and Sirt1 is capable of deacetylating Piezo1. (Figure 4B,C). Co‐IP experiments revealed that Sirt1 bound to Piezo1 (Figure 4D,E). SRT2104(SRT), a specific activator of Sirt1, reduced the acetylation of Piezo1 (Figure 4F). Moreover, we found that both overexpression and pharmacological activation of Sirt1 dramatically increased the level of Piezo1 protein in a dose‐dependent manner (Figure 4G,H). Based on the subcellular colocalization analysis technique in light microscopy,^[^ ^22^, ^23^ ^]^ IF imaging revealed that colocalization of Piezo1 and Sirt1 (Figure 4I,J). We determined the effect of SRT on Piezo1‐mediated calcium flow. The results showed that both SRT and Yoda1 similarly induced cellular calcium flow, both of which was dramatically lower in calcium‐free ringer solution than those in calcium‐containing ringer solution (Figure 4K,L), suggesting that significant amount of the calcium flow induced by either activator is through the cell membrane. Importantly, siRNA knockdown of Piezo1 expression dramatically reduced SRT‐ or Yoda1‐induced elevations in calcium flow (Figure 4M,N). Furthermore, the increase of calcium flow induced by SRT, but not Yoda1, was inhibited by 3‐TYP (a Sirt deacetylase inhibitor), but not by Panobinostat (a pan‐HDAC deacetylase inhibitor) (Figure 4O‐Q), suggesting that the Sirt rather than HDAC pathway is involved in SRT activation of Piezo1. Next, we constructed the plasmid L1341K/L1344K with mutations at key sites of Piezo1 mechanical force transduction^[^ ^24^ ^]^ and found that Yoda1 no longer activated Piezo1 calcium flow in cells transfected with L1341K/L1344K plasmid, while these Piezo1 mutations had no marked effect on SRT activated calcium flow (Figure 4R). These results suggest that the calcium flow caused by Piezo1 deacetylation is not via the conventional mechanical stress. We predicted the interaction between Piezo1 and Sirt1 and the structure of the complex using AlphaFold 2 and visualized it with ChimeraX, which demonstrated that Sirt1 forms stable hydrogen bonds with the lysine residues K1525 and K2174 in Piezo1. K1525, located near the beam, forms three hydrogen bonds with Sirt1, while K2174 is situated in the C‐terminal region, forms one hydrogen bond with Sirt1. Both the beam and C‐terminus are important for mechanical transduction of Piezo1 (Figure S3A, Supporting Information). We next determined whether Sirt1 can deacetylate these specific lysine residues in Piezo1. Through multiple sequence alignment analyses, we found that K2174 of Piezo1 is highly conserved across different species, while K1525 shows relatively poor conservation (Figure S3B, Supporting Information). In protein acetylation studies, lysine mutations to arginine can maintain deacetylation, whereas mutations to glutamine preserve a neutral state. Next, we performed structural superposition and sequence alignment of Piezo1 with lysine mutations at both sites or Sirt1 intervention using ChimeraX 1.18. The results indicated that Sirt1 binding to Piezo1 can induce spatial conformational changes in the central pore (Figures S3C and S4, Supporting Information). After the K1525R/Q mutations, significant conformational changes were observed in the central pore of Piezo1. K1525Q, when combined with Sirt1, also led to noticeable changes in the central pore's conformation. In contrast, no significant conformational changes were observed in Piezo1 after Sirt1 intervention alone, suggesting that the K1525Q mutation does not affect Sirt1's influence on Piezo1 activity, indicating that K1525 may not be the key site for Sirt1 modulation of Piezo1 activation (Figures S3C and S4, Supporting Information). Following the K2147R mutation, a significant change in the central pore's spatial conformation was observed in Piezo1, whereas no significant change occurred after K2174Q mutation. Moreover, when K2174Q was combined with Sirt1 intervention, only minor conformational changes were observed compared to Piezo1 or Piezo1 with Sirt1 intervention alone. This suggests that K2174 may be the key site for Sirt1‐mediated activation of Piezo1(Figures S3C and S4, Supporting Information).

In addition, our results suggest that Piezo1 activation suppresses Sirt1 expression (Figure S5A, Supporting Information). These findings may reflect the complex regulatory mechanisms underlying fracture healing.

### 2.5 Oral YC‐SRT is Highly and Selectively Targeted to the Fracture Site in Mice

**Figure 5:**
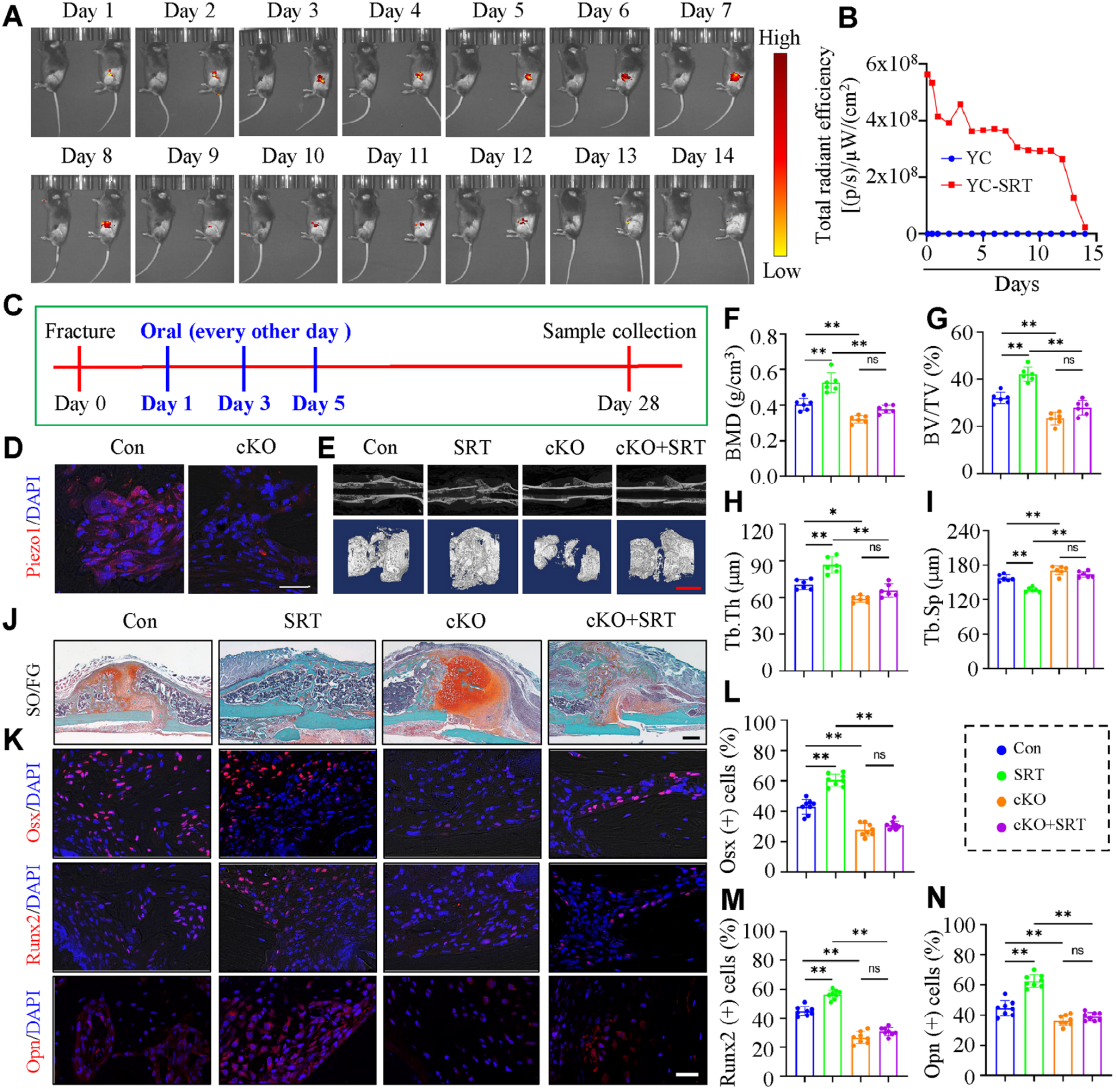
Oral YC‐SRT is highly and selectively targeted to the fracture site in mice. A,B) In vivo fluorescence imaging (A) and fluorescence intensity quantification (B) at different time points after oral delivery of YC‐SRT (N = 3). C) Schematic diagram illustrating the experimental design. D) Representative images of IF staining of Piezo1 in callus of control and cKO mice. Scale: 50 µm. E) Representative X‐ray images and 3D reconstruction of µCT scans of callus in two groups of mice treated with YC and YC‐SRT at 4 weeks after fracture surgery. Scale bar: 1.0 mm. F‐I) The BMD (F), BV/TV (G), Tb.Th (H), and Tb.Sp (I) of callus of the callus were analyzed based on µCT results in (E). J) Representative images of SO/FG staining of callus of each group mice at 4 weeks after fracture surgery. Scale bar: 200 µm. K) Representative images of IF staining of Osx, Runx2 and Opn in callus of each group mice at 4 weeks after fracture surgery. Scale bar: 50 µm. L‐N) Quantification of Osx, Runx2 and Opn positive cells in callus based on the IF staining results in (K). N = 6 per group. Results are expressed as mean ± standard deviation (s.d.). **P < 0.01.

Given the critical role of Sirt1 in activating Piezo1 as demonstrated above, we further investigated whether Piezo1 is required for Sirt1 promotion of bone healing. SRT2104 (SRT) is a highly specific Sirt1 activator that has been demonstrated to have favorable therapeutic effects on various conditions, such as neurodegenerative diseases, cardiovascular diseases, musculoskeletal diseases, and other diseases.^[^ ^25^ ^]^ The therapeutic use of SRT is restricted in part due to its poor solubility in water, which largely limits its bioavailability. Oral administration is the most widely used route to treat various diseases due to its convenience, high patient acceptance, cost‐effectiveness, and high safety.^[^ ^26^ ^]^ To overcome the limitations of traditional oral formulations, such as short residence time in the digestive system, limited targeting ability, and water insolubility and low bioavailability of biologic drugs, based on our previous studies,^[^ ^21^ ^]^ we have developed a yeast drug delivery platform that can be orally administrated and selectively targeted to the inflammatory fracture sites as a high effective payload and sustainable release for fracture treatment. The oral YC‐SRT was constructed in three main sequential modification steps: cationic nanoparticles (NP) synthesis, NP self‐deposition, and surface enteric coating (Figure S6A, Supporting Information). Initially, yeast microcapsules (YCs) with ≈5.0 µm in diameter were purified from S. cerevisiae by acidic and alkaline extraction to remove the cytoplasm and cell‐wall polysaccharides. Next, cationic PEI‐SRT NPs were prepared by self‐assembly of bPEI and SRT, and their shape, size and ζ‐potentials were determined using SEM and dynamic light scattering (DLS), respectively (Figure S6B‐F, Supporting Information). To accomplish the drug loading to YCs, cationic PEI‐SRT NPs were loaded into negatively charged YCs by electrostatic deposition. Then, the YC‐SRT was characterized by confocal fluorescence imaging using cyanine (Cy5.5)‐labeled SRT. The merged images verified that PEI‐SRT loaded cationic nanoparticles were efficiently packaged into YCs (Figure S6G, Supporting Information). The encapsulation efficiency of the YC‐SRT was 50.9%, and the loading contents of the YC‐SRT was 31.7 µg mg^−1^ (Figure S6H, Supporting Information). These results indicate that YCs are high‐efficient carriers for SRT. We further determined the drug release curves of YC and YC‐SRT at pH 6.6 (simulating the acidic microenvironment of the fracture site) and pH 7.4 (simulating the normal bone microenvironment), respectively. YC‐SRT exhibited a relatively stable drug release (≈6%) within 100 h at pH 7.4. In acidic environments (pH 6.6), it exhibited a more rapid and effective drug release (≈12%) (Figure S6I, Supporting Information). We next determined the effect of YC‐SRT on fracture healing in mice. YC‐SRT were given orally post operation and analyzed 4 weeks post operation. YC‐SRT did not affect the weight and histology of major organs in mice (Figure S7, Supporting Information). Importantly, the fluorescence signal of YC‐SRT was detected only at the fracture site, which peaked on the 7th day and maintained for at least 14 days (**Figure** **5**A,B). These results demonstrate that targeted delivery of SRT to the fracture site via oral yeast microcapsules has high efficiency and sustained release.

### 2.6 Piezo1 Deficiency Attenuates Sirt1‐Accelerated Fracture Healing in Mice

We next utilized our novel deliver system developed above to determine the effect of Sirt1 activation on fracture healing in mice; if so, does this require Piezo1 in chondrocytes. To this end, control and cKO mice were randomly divided into 2 groups for fracture surgery and intervention, and samples were collected 4 weeks later (Figure 5C,D). Results showed that YC‐SRT increased in the callus mineralization, bone mineral density, trabecular thickness and density, and the expression levels of osteogenic genes, when compared to those in untreated control mice. Piezo1 loss reduced the values of all above parameters (Figure 5E‐I). Importantly, the effect of SRT on promoting fracture healing was largely attenuated by Piezo1 deficiency in mice (Figure 5E‐I). At the molecular level, Sirt1 activation increased expression of Runx2 and Osx, which was abolished by Piezo1 deficiency (Figure 5J‐N).

### 2.7 YC‐RSV Promotes Callus Mineralization in Mice

**Figure 6:**
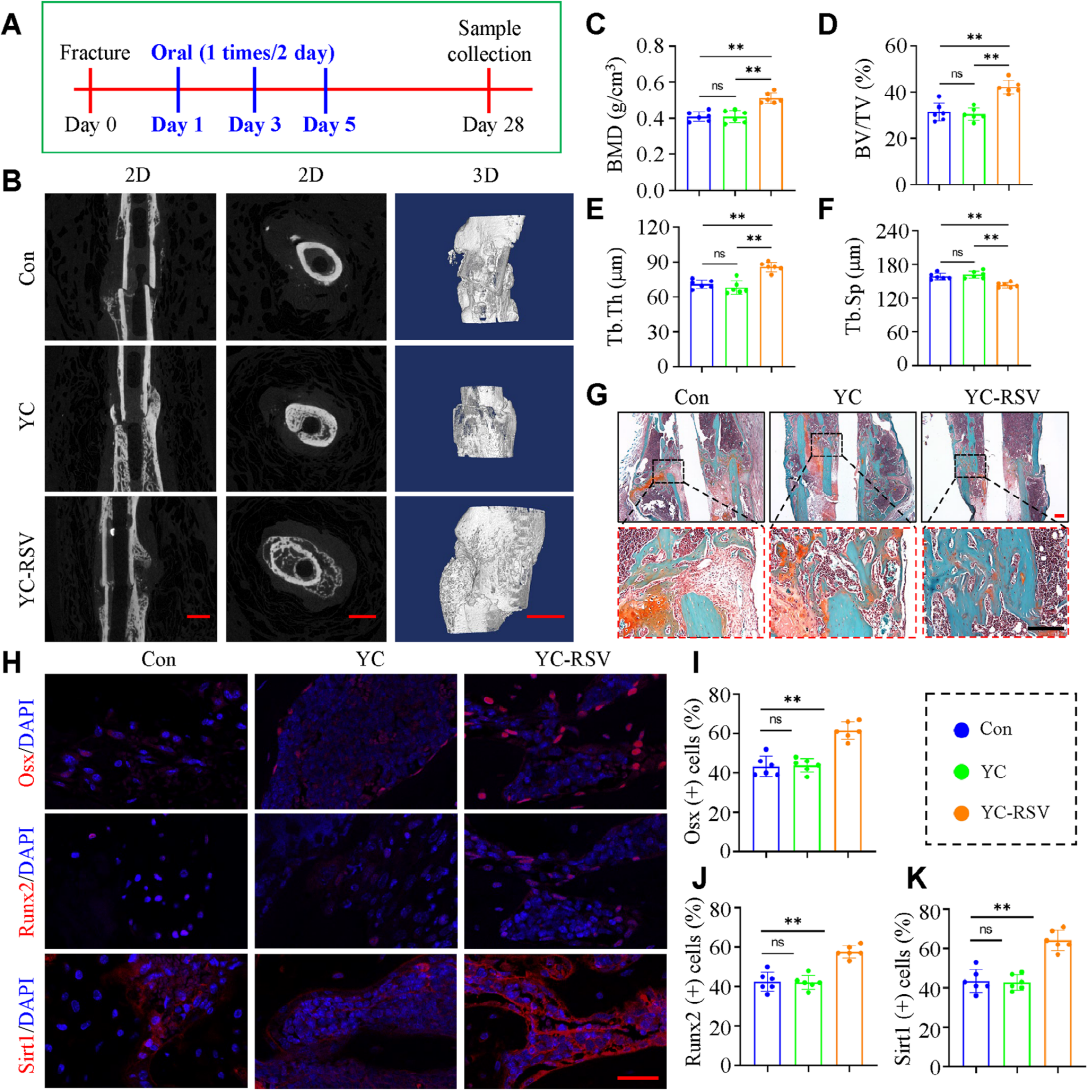
YC‐RSV promotes callus mineralization in mice. A) Schematic diagram of the in vivo experimental design. B) Representative X‐ray images and 2D/3D reconstruction of µCT scans of mice callus in Con, YC, and YC‐RSV groups at 4 weeks after fracture surgery. Scale bar: 1.0 mm. C‐F) The BMD (C), BV/TV (D), Tb.Th (E) and Tb.Sp (F) of the callus were analyzed based on µCT results in (B). G) Representative images of SO/FG staining of callus in three groups of mice at 4 weeks after fracture surgery. Scale bar: 200 µm. H) Representative images of IF staining of Osx, Runx2 and Sirt1 in callus of each group mice at 4 weeks after fracture surgery, Scale bar: 50 µm. I‐K) Quantification of Osx, Runx2 and Sirt1 positive cells in callus based on the IF staining results in (H). N = 6 per group. Results are expressed as mean ± standard deviation (s.d.). **P < 0.01.

Resveratrol (RSV) is a natural activator of Sirt1, and its efficacy, safety and pharmacokinetics have been well documented in more than 244 clinical trials.^[^ ^27^ ^]^ In view of the ameliorative effect of RSV on bone mass loss, we first examined the intervention effect of RSV on bone healing. Due to the rapid metabolism and poor bioavailability of RSV, which limits its therapeutic use,^[^ ^27^ ^]^ we developed a yeast microcapsule‐based drug delivery platform that can be orally absorbed and targeted at fracture sites, which is closer to clinical application. Oral YC‐RSV was constructed as described in Figure S6J (Supporting Information). Briefly, cationic PEI‐RSV NPs were firstly prepared by self‐assembly of bPEI and RSV, and their shape, size and ζ‐potentials were determined (Figure S6K‐O, Supporting Information). The cationic PEI‐RSV NPs were then loaded into negatively charged YCs by electrostatic deposition. Afterward, the YC‐RSV was characterized by confocal fluorescence imaging showing that PEI‐RSV loaded cationic nanoparticles was efficiently packaged into YCs (Figure S6P, Supporting Information). The encapsulation efficiency of the YC‐RSV was 73.1%, and the loading contents of the YC‐RSV was 51.2 µg mg^−1^ (Figure S6Q, Supporting Information). YC‐RSV exhibited relatively stable drug release (≈7%) within 100 h at pH 7.4. In acidic environments (pH 6.6), it exhibited more rapid and effective drug release (≈13.5%) (Figure S6R, Supporting Information). Finally, we determined the effect of RSV on fracture healing in mice. YC‐RSV was given orally post operation and analyzed 4 weeks post operation. Neither YC nor YC‐RSV displayed any detrimental effects on major organs, including heart, liver, spleen, lung and kidney (Figure S7, Supporting Information). While YC alone had no significant effect on fracture healing, YC‐RSV promoted fracture healing by promoting the callus mineralization and largely increased osteogenesis across the fracture space (**Figure** **6**A‐G). RSV treatment increased expression of Osx and Runx2 in the callus in mice (Figure 6H‐J). Notably, RSV also elevated the *Sirt1* expression in the callus (Figure 6H,K).

### 2.8 RSV@GelDa Promotes Bone Formation and Repair in Mice

**Figure 7:**
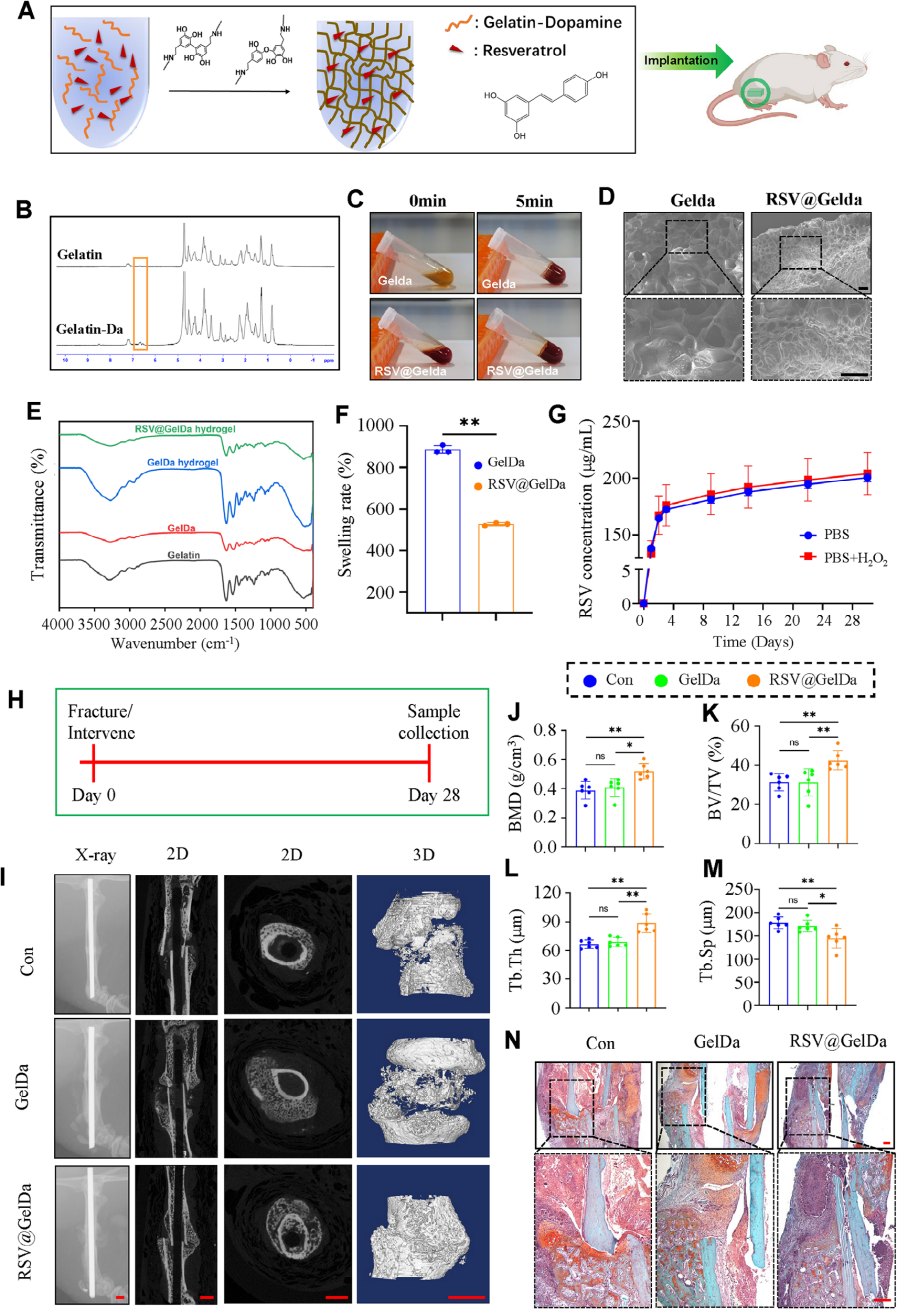
RSV@GelDa promotes bone formation and repair in mice. A) Schematic diagram showing the fabrication and administration of RSV@GelDA hydrogel. B) 1H nuclear magnetic resonance (1H NMR) spectra of Gelatin and Gelatin‐Da, with the dopamine‐specific peaks of 6.5–7 ppm in the orange box. C) Overview of hydrogel formation processes in GelDa (up) and RSV@GelDa (down). D) Scanning electron microscope (SEM) images of GelDa (left) and RSV@GelDa inner sections (right). Scale bar: 100 µm. E) Detection of surface functional groups of these materials by the fourier transform infra‐red (FTIR). F) Swelling rate of freeze‐dried GelDa and RSV@GelDa hydrogels, N=3 per group. G) Drug release curves of RSV@GelDa hydrogel in PBS and ROS environments (37 °C), N = 3 per group. Results are expressed as mean ± standard deviation (s.d.). **P < 0.01. H) Schematic diagram of the in vivo experimental design. I) Representative X‐ray images and 2D/3D reconstruction of µCT scans of mice callus in Con, GelDa, and RSV@GelDa groups at 4 weeks after fracture surgery. Scale bar: 1.0 mm. J‐M) The bone mineral density (BMD) (J), bone volume/tissue volume (BV/TV) (K), trabecular thickness (Tb.Th) (L), and trabecular separation (Tb.Sp) (M) of the callus were analyzed based on µCT results in (I). N) Representative images of SO/FG staining of callus in three groups of mice at 4 weeks after fracture surgery. Scale bar: 200 µm. N = 6 per group. Results are expressed as mean ± standard deviation (s.d.). **P < 0.01.

In view of the promoting effect of oral administration of RSV on fracture healing, we finally examined the intervention effect of local administration of RSV into the fracture site on fracture healing. We developed an injectable drug loaded hydrogel platform as a high payload and sustainable release for fracture treatment. In the presence of H_2_O_2_ and HRP, the oxidative polymerization of RSV with catechol groups in Gelda molecules formed RSV@Gelda (gelatin‐dopamine hydrogel‐based delivery system loaded with resveratrol) (**Figure** **7**A). We first synthesized Gelda by grafting dopamine groups onto the gelatin skeleton. 1H nuclear magnetic resonance (1H NMR) observed a specific peak of 6.5–7 ppm in Gelda and dopamine, but not in the gelatin spectrum, indicating that dopamine was successfully grafted onto the gelatin skeleton (Figure 7B). As shown in Figure 7C, Gelda remained liquid after dissolution and flowed when tilted. It formed a gel after brief mixing with HRP and H_2_O_2_, and did not flow when tilted (Figure 7C). Results from SEM revealed that both RSV@Gelda and Gelda hydrogels showed porous structures, but RSV@Gelda had a denser microscopic pore structure (Figure 7D). We then analyzed the surface functional groups of the hydrogels by FTIR spectroscopy. For all hydrogels, peak values were observed at 3270 cm^−1^ (phenol O‐H stretching) and 1245 cm^−1^ (amine C‐N stretching). At 1155 cm^−1^ band, phenolic compounds containing carbonyl group was found. The formation of the (HN‐CO) bond was shown at 1631 cm^−1^ band, confirming that dopamine forms a chemical bond with gelatin^[^ ^28^, ^29^ ^]^ (Figure 7E). The freeze‐dried RSV@Gelda hydrogel significantly reduced the swelling rate compared with GelDa hydrogel, indicating that RSV crosslinking promoted the hydrogel to have a denser internal structure (Figure 7F). Lines of evidence have shown that ROS is overproduced in bone defect region.^[^ ^30^ ^]^ To simulate the release environment of hydrogels in vivo, in addition to detecting the RSV release efficiency of hydrogels in PBS, we also studied the effect of ROS on the RSV release of hydrogels. Sustained drug release experiments showed that RSV@Gelda hydrogel could slowly release RSV for up to 28 days without being affected by H_2_O_2_ (Figure 7G).

We finally determined the effect of local administration of the RSV@Gelda on fracture healing in mice. Gelda and RSV@Gelda were locally injected to the fracture site during the operation and analyzed 4 weeks after operation. Both Gelda and RSV@Gelda did not exhibit any organ and cell toxicity (Figure S8, Supporting Information). The hydrogel alone had no marked effect on fracture healing. Importantly, RSV@Gelda promoted expression of osteogenic proteins and fracture healing by promoting the callus mineralization and increasing trabeculae volume across the fracture gap in mice (Figure 7H‐N).

## 3 Discussion

**Figure 8:**
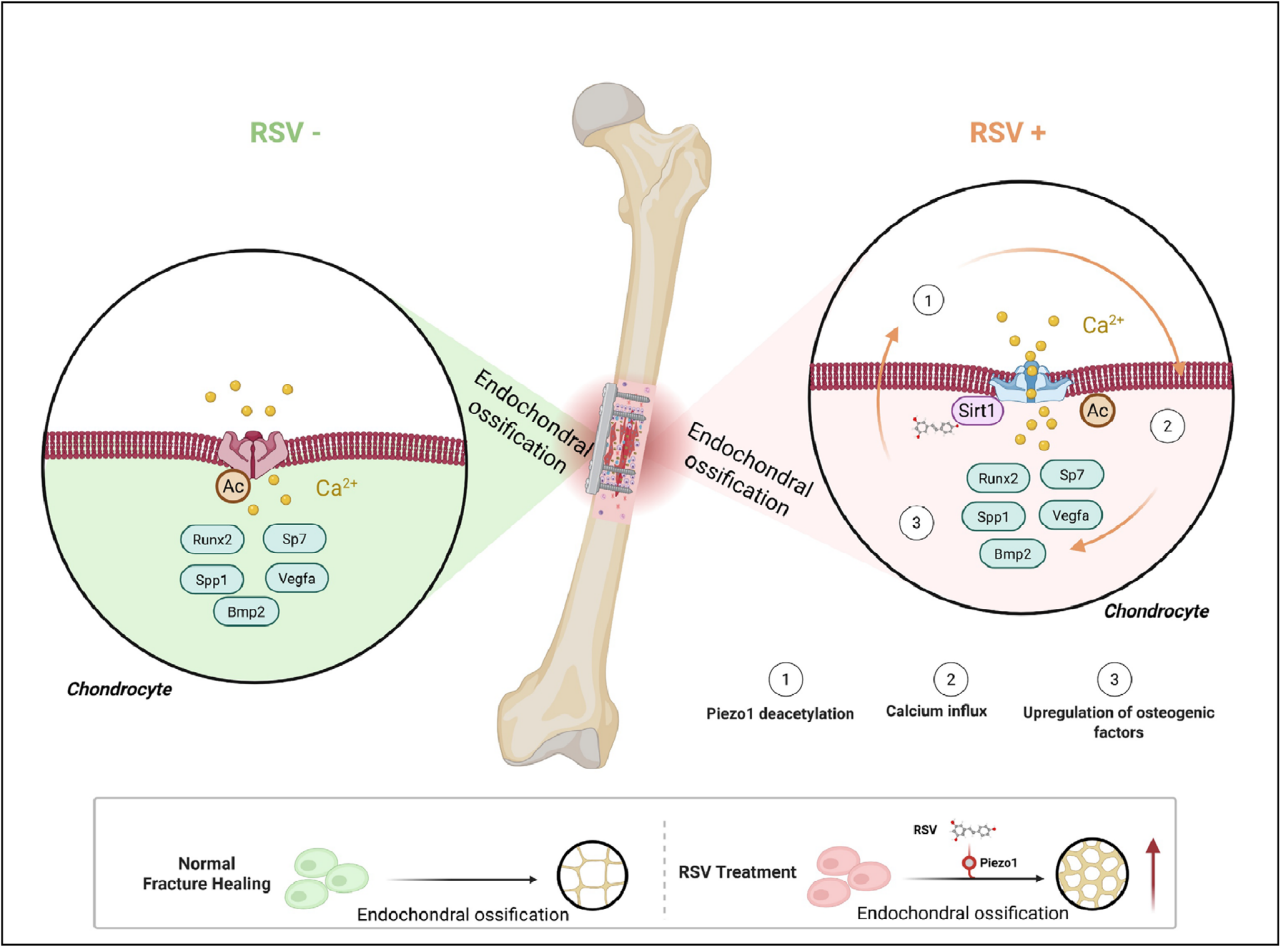
Schematic of the Sirt1–Piezo1 regulatory axis and its function in fracture healing. Left panel: Under physiological conditions during fracture healing, Piezo1 functions in endochondral ossification. Right panel: After RSV (resveratrol) intervention, deacetylation of Piezo1 results in its activation, which upregulates both osteogenic and angiogenic factors, thereby promoting endochondral ossification. Steps 1, 2, and 3 indicate the sequential stages of RSV‐enhanced fracture healing. “Ac” denotes acetylation. RSV refers to resveratrol.

In this study, we demonstrate a crucial role of deacetylation activation of Piezo1 in promoting osteogenesis and fracture healing in mice (**Figure** **8**). A significant upregulation of Piezo1 expression in chondrocytes in human and murine cartilage callus suggests that Piezo1 is intrinsically involved in bone repair. This notion is supported by evidence from experiments using multiple genetic mouse models. Our results are consistent with those from recent studies showing the importance of Piezo1 and SIRT1 in bone repair.^[^ ^31^, ^32^ ^]^ Notably, our study extends these findings by employing a distraction osteogenesis model. We further demonstrate that the activation of Sirt1 promotes the deacetylation of lysine in Piezo1 molecule, leading to Piezo1 activation, Alphafold2 prediction suggested that K2174 may be a critical site for Sirt1‐mediated activation of Piezo1. By using a novel drug delivery system through oral administration and fracture site targeting, we demonstrate that SRT, a specific Sirt1 activator, greatly accelerates bone healing in mice, which requires the presence of Piezo1 expression in chondrocytes. Based on the idea of new application of old drugs,^[^ ^33^ ^]^ we identify RSV, a natural Sirt1 activator, as a potent Piezo1 activator and demonstrate that it promotes bone healing via either oral or local administration.

Notably, the combination of the classical bone fracture model and a unique DO model with Piezo1 transgenic mice provides an opportunity to study the role of Piezo1 in endochondral ossification during bone healing. The gene expression in chondrocytes is consistent with that from our previous studies on the role of Piezo1 in the pathogenesis of osteoarthritis in chondrocytes.^[^ ^34^ ^]^ Our results reveal that Piezo1 deficiency in chondrocytes decreases the expression of osteogenic proteins, and delays fracture healing and DO process, which may be related to the inhibition of endochondral ossification during bone healing. The deficiency of Piezo1 in chondrocytes was shown to impair endochondral ossification and the formation of bone trabeculae and secondary spongy tissue.^[^ ^35^ ^]^ In this study, we find that Piezo1 loss in the chondrocytes results in thickening of the proliferative zone of the growth plate, which may be involved in promoting chondrocyte proliferation and inhibiting chondrocyte transdifferentiation or redifferentiation. In addition, Piezo1 loss in chondrocytes significantly reduces expression of focal adhesion proteins after FSS loading, and our previous studies showed that Kindlin‐2 loss in chondrocytes led to defective chondrocyte differentiation and impairment of endochondral ossification.^[^ ^36^ ^]^ This may in part explain why Piezo1 loss in chondrocytes impairs endochondral ossification during fracture healing and DO process. In addition to the effect on osteogenesis, our results from the study show that Piezo1 loss in chondrocytes promotes osteoclast activity near the growth plate, which is inconsistent with the findings of Brylka LJ et al., who showed in their studies that *Piezo1; Col2a1^Cre^* mice displayed a widening of the growth plate after birth compared to the control group, which was attributable to an extension of the proliferative zone, but was not accompanied by an increase in TRAP staining below the growth plate.^[^ ^37^ ^]^ Wang L et al. observed increased TRAP staining in the tibia in *Piezo1; Prx1^Cre^* mice,^[^ ^18^ ^]^ which is consistent with our findings. This discrepancy may be related to distinct cell populations targeted by different transgenic Cre lines used in these studies. Novak S et al. reported that *Aggrecan^CreER^* lacked specificity for chondrocytes in fracture callus, but it could also target periosteal progenitor cells in fracture callus.^[^ ^38^ ^]^ While these results may partially explain why Piezo1 loss in chondrocytes inhibits endochondral ossification during fracture and DO process, they also suggest the presence of complicated mechanism(s) underlying Piezo1 regulation of the endochondral ossification during fracture healing.

Previous studies on the regulation of Piezo1 activity primarily focus on the direct binding of the partner proteins, with limited information currently available on the post‐translational regulation of Piezo1 activity. In the present study, we demonstrate that the deacetylation‐acetylation of Piezo1 is an important regulation of the activation and closure of the ion channel, which is different from the channel opening mechanism caused by Yoda1 or mechanical stress. As a large ion channel protein, Piezo1 can change its structure and affect channel opening due to membrane tension or mechanical stress at the distal lever.^[^ ^39^ ^]^ It is possible that SRT or RSV induction of Piezo1‐dependent calcium flow is due to a conformational change of Piezo1 protein caused by deacetylation, resulting in opening of the ion center pore. Notably, activating Sirt1, rather than HDAC, promotes the deacetylation and activation of Piezo1. Furthermore, our findings suggest that Sirt1 facilitates the upregulation of Piezo1 probably through a mechanism involving Sirt1‐mediated deacetylation and subsequent stabilization of Piezo1. Thus, we add a new layer of regulation of Piezo1 activity.

In this study, we have developed two novel oral formulations, YC‐RSV and YC‐SRT, and, for the first time to our knowledge, applied this yeast‐based delivery system to the treatment of bone fractures. Our findings reveal that both formulations enhance fracture healing in mice. Advantages of this novel deliver system are obvious. It can be orally administrated, which provides high patient compliance and convenience. Many small molecule compounds, including SRT, are characterized by low solubility, rapid metabolism and poor targeting of systemic administration. According to our previous studies, oral drug‐loaded YC can be absorbed into intestinal lymph tissues through transcytosis of microfold cells, rapidly transmigrated to the spleen via lymphatic circulation, phagocytosed by macrophages, and then transmigrated from the spleen and rapidly homed to inflamed sites through the blood circulation. In our study, we have utilized the yeast capsule (YC) system not only for sustained drug delivery but also for its ability to exploit macrophage trafficking and intracellular dynamics. YC particles can be retained within the lysosomes of macrophages for extended periods. Upon inflammatory stimulation, the encapsulated drug is released from the lysosomal compartment into the cytoplasm and subsequently secreted into the extracellular environment via macrophage exocytosis.^[^ ^21^ ^]^ Importantly, the inflammatory phase of fracture healing occurs within the first 1–7 days post‐injury, characterized by local hemorrhage and the recruitment of inflammatory cells, such as macrophages and monocytes. This window provides a critical opportunity for YC‐mediated delivery to target the early inflammatory niche and modulate the formation of cartilage callus. In addition, even large amounts of oral YC are safe and non‐toxic to the body.^[^ ^40^ ^]^ In this study, we have constructed a long‐acting, sustained‐release, non‐toxic yeast drug delivery platform that can be absorbed orally, and highly and selectively targeted to the inflammation site of fractures by macrophage‐mediated inflammatory migration. Although the release rate of the YC delivery system is not very high, it provides a sustained and prolonged drug release over a period of 1 to 14 days, which aligns well with the extended timeline required for fracture healing. This sustained release ensures continuous local exposure to the therapeutic agents during the critical phases of bone repair. Furthermore, oral YC‐SRT also promotes fracture healing in mice, which was largely attenuated by Piezo1 deletion in chondrocytes. Notably, the YC‐SRT fluorescence signal was exclusively localized to the fracture site and persisted for at least 14 days, demonstrating that the yeast microcapsule‐based oral delivery system exhibits both high efficiency and sustained‐release properties. It is known that RSV regulates multiple cellular signaling pathways in a Sirt1‐dependent manner.^[^ ^41^ ^]^ RSV has various pharmacological activities, including antioxidant and anti‐inflammatory activities, which may influence fracture healing through multiple pathways.^[^ ^27^ ^]^ However, prior studies^[^ ^42^, ^43^ ^]^ and our pharmacological and expression data suggest that its anti‐inflammatory effects are at least partially mediated through the Sirt1–Piezo1 axis. While we cannot completely exclude other mechanisms, our findings provide strong evidence supporting a central role for this pathway in RSV‐mediated bone repair. Our in vivo studies further confirm that oral administration of YC‐RSV significantly increases the expression of osteogenic proteins, which contributes in part to its fracture healing‐promoting effect. Meanwhile, oral administration of YC‐RST/RSV induced no significant pathological alterations or abnormal body weight changes in mice, confirming the favorable biosafety profile of yeast microcapsule‐based oral delivery system.

In summary, our study uncovers pivotal novel findings regarding the role and mechanism through which the mechanically sensitive cation channel Piezo1 mediates mechanically induced osteogenesis and promotes bone fracture healing, as well as relevant potential translational significance of our findings. Crucially, we demonstrate that resveratrol (RSV), a natural Sirt1 activator, functions as a potent Piezo1 agonist capable of enhancing murine bone repair. Furthermore, we engineered an oral drug‐delivery platform with optimal safety and efficacy profile, enabling targeted Piezo1 activation at fracture sites, thereby establishing a novel precision therapeutic paradigm with significant translational implications for clinical bone repair.

## 4 Experimental Section

### Single Cell Analysis

To characterize gene expression at single cell level, two published single cell RNA sequencing (scRNA‐seq) dataset, GSE150291 and GSE154247 were investigated.^[^ ^44^, ^45^ ^]^ The raw sequencing data at FASTQ format was downloaded, and was subsequently processed with Cell Ranger for expression matrices. For RNA velocity, cells of MSC, OLC, and chondrocyte were adopted in scVelo analysis.

### Human Tissue Specimens

Six patients who underwent internal fixation surgery for fractures at the Affiliated Hospital of Shandong University of Traditional Chinese Medicine 2‐6 weeks after the fracture due to displacement or non‐union of the fracture, callus tissues and paired bone tissues excised during surgery were collected, and the information of the specimens was shown in Table S2 (Supporting Information). All specimens obtained patient consent and approval from the Medical Ethics Committee of the Affiliated Hospital of Shandong University of Traditional Chinese Medicine (2024‐165‐KY).

### Mice

*Piezo1^flox/flox^* mice were purchased from the Jackson Laboratory (Bar Harbor, ME, USA). *Aggrecan^CreERT2^* mice was previously described.^[^ ^34^ ^]^ Both mice were in stable C57BL/6 background for more than ten generations. It crossed *Piezo1^flox/flox^* mice with *Aggrecan^CreERT2^* mice to generate *Piezo1^flox/flox^; Aggrecan^CreERT2^* mice to delete Piezo1 in chondrocytes as previously described.^[^ ^46^ ^]^ In vivo studies were conducted on 12‐week‐old mice. *Piezo1^flox/flox^; Aggrecan^CreERT2^* mice were intraperitoneally injected with tamoxifen (TAM, 1 mg/10 g body weight; Sigma–Aldrich) 4 days after fracture surgery or 7 days after DO surgery^[^ ^47^, ^48^ ^]^ for 5 consecutive days to delete Piezo1 in chondrocytes,^[^ ^49^ ^]^ which as conditional knockout (cKO) group. *Piezo1^flox/flox^* mice were used as the control group, and an equal amount of TAM were injected at the same time. To minimize animal use and experimental variability,^[^ ^50^, ^51^ ^]^ male mice was used in this study to avoid confounding effects of estrogen fluctuations on fracture healing, and all mice were housed under standard conditions in the specific pathogen free (SPF) experimental animal center of the Southern University of Science and Technology (SUSTech). All animal experiments were approved by the Institutional Animal Care and Use Committee (IACUC) of SUSTech (SUSTech‐JY202203002‐202408A1). All relevant guidelines for mouse experiments were adhered to in this study.

### Preparation of Yeast Microcapsules (YCs) and Cationic Nanoparticles (NPs)

Baker's yeast (S. cerevisiae) was provided by Leadfree Management Co. Ltd. (Shanghai, China). Resveratrol (RSV) and SRT2104 (SRT) were obtained from the MedChemExpress (MCE). The commercial Cy5‐NHS ester and branched polyethyleneimine (bPEI, molecular (weight = 25 kDa) were purchased from Sigma–Aldrich (Missouri, USA). All other chemicals and reagents used were of analytical grade. Hollow YCs were purified from baker's yeast through alkaline and acid extraction methods.^[^ ^52^ ^]^ As our previously described,^[^ ^21^ ^]^ baker's yeast was dissolved in 1 M sodium hydroxide (NaOH), and the solution was heated at 80 °C for 1 h. After centrifugation at 3000 g for 10 min, the pellet was rinsed twice with deionized water. Then, the sample was dispersed in hydrochloric acid (HCl) solution at pH 4.0 and in curated at 60 °C for 1 h. Subsequently, the obtained sample was rinsed with isopropyl alcohol and acetone, and the YCs were collected and dried at room temperature. Cationic NPs were prepared according to the self‐assembly method described previously.^[^ ^53^ ^]^ In short, RSV or SRT was mixed with bPEI in anhydrous dimethyl sulfoxide (DMSO) at a weight concentration of 10 mg mL^−1^ for 2 h, and then dialyzed with deionized water at 37 °C for 24 h to obtain the PEI‐SRT/RSV cationic nanoparticles.

### Preparation and Characterization of YC‐RSV/SRT

First, 10 mg of YCs and 100 µL of PEI‐RSV/SRT solution were resuspended in deionized water. The suspension was poured into a 5 mL centrifuge tube and heated in a 37 °C water bath for 2 h to obtain the YC‐RSV/SRT. After the reaction, unloaded PEI‐RSV/SRT was removed by centrifugation and washing with deionized water. The PEI‐RSV/SRT was cleaned up again after overnight at 4 °C. Considering oral administration, glycerin was added to YC‐RSV/SRT for the purpose of lubricating the esophagus. To further identify of YC‐RSV/SRT, SEM and TEM images of the YC‐RSV/SRT were obtained using SUPRA55 instrument (Zeiss, Germany) and JEM‐1400Plus microscope (JEOL, Japan). RSV or Cy5.5 probe labeled SRT was imaged and analyzed using a STELLARIS 5 confocal microscope (LEICA, Germany) to further verify that RSV/SRT was successfully loaded into YCs. Meanwhile, the particle size and zeta potential of YC‐RSV or YC‐SRT as well as PEI‐RSV and PEI‐SRT particles were determined using a using Malvern Zetasizer Nano ZS instrument. Next, the drug release profile of YC‐RSV/SRT was evaluated in a simulated normal(pH6.6) and fracture site microenvironment^[^ ^54^, ^55^, ^56^ ^]^ (pH 7.4) added with 0.2% Triton X‐100 surfactant. The concentration of SRT and RSV in the buffer was quantified by fluorescence spectroscopy and UV spectrophotometry, respectively.

### Synthesis and Characterization of Dopamine‐Grafted Gelatin

Gelatin‐Dopamine (Gelda) was synthesized as previously described.^[^ ^57^ ^]^ First, 10 g of gelatin (Sigma–Aldrich) was dissolved in 1 L of deionized water at 40 °C until it was completely dissolved under N_2_. N‐(3‐dimethylaminopropyl)‐N’‐ethylcarbodiimide hydrochloride (EDC, Aladdin) (4 g, 20.87 mmol), N‐hydroxysuccinimide (NHS, Aladdin) (4 g, 34.76 mmol), and dopamine hydrochloride (Sigma–Aldrich) (6 g, 31.64 mmol) were added to the gelatin solution successively, and the chemical reaction was carried out for 6 h at 50 °C and pH ≈5. Then the reaction solution was put into a dialysis bag (MWCO = 8000–14000 Da) and immersed in aqueous solution of NaCl (20 × 10^−3^ M) and HCl (833 × 10^−6^ M) at 40 °C for 3 days to remove by‐products such as EDC, NHS and dopamine with molecules smaller than 8 kDa, and then soaked in deionized water for 3 h to remove NaCl. The purified Gelda was collected and freeze‐dried for 3–5 days, and the final product was stored at −20 °C. The molecular structure of Gelda was confirmed by ^1^H‐NMR (Bruker Ascend 600).

### Fabrication and Characterization of RSV@Gelda Hydrogel

At 37 °C, Gelda (150 mg) was dissolved in PBS (0.7 mL) and then added to resveratrol (RSV) (0.1 mL) solution for polymerization. Then the polymer solution was fully mixed with HRP(Aladdin) (0.1 mL, 3.2 mg·mL^−1^) solution and H_2_O_2_ solution (0.1 mL, 1–5 wt.%) and stood for hydrogel crosslinking.^[^ ^58^ ^]^ The gelation time was determined by vial tilting method. The final concentrations of Gelda, HRP, H_2_O_2_ and RSV were 15 wt.%, 0.32mg·mL^−1^, 0.1–0.5 wt.% and 1–10 mM, respectively. The structures and compositions of the hydrogels were detected by Fourier transform infrared spectroscopy (FTIR, Thermo‐Nicolet Nexus 670 ATR‐IR spectrometer). The hydrogel samples were prepared and swollen to saturation, then freeze‐dried and sliced for microstructure analysis. Field emission SEM and energy dispersive X‐ray spectroscopy were used to observed and photographed the microstructure of Gelda hydrogel and RSV@Gelda hydrogel after sputtering gold. The freeze‐dried hydrogel samples were weighed and recorded as *W*_0_, and then immersed in PBS water at 37 °C for 24 h, using filter paper to remove excess surface moisture and the weight of each swelling sample was measured and recorded it as *W*_t_. The swelling capacity of the hydrogel was assessed as: Swelling ratio = (*W*_t_ ‐*W*_0_)/ *W*_0_ × 100 %. To test the release time‐concentration curve of RSV, 0.2 mL RSV@Gelda hydrogel was soaked in 2 mL PBS and placed it on a shaker at 37 °C. At the scheduled time, PBS extracts were collected and replaced with fresh PBS for sustained release. The concentration of RSV in solution was determined by High Performance Liquid Chromatography (HPLC).

### Femoral Fracture Model

The surgery was performed as previously described.^[^ ^59^ ^]^ Briefly, after anesthetizing the mice, the left femur was sterilized, then a transverse fracture was formed at the mid‐shaft with a wire saw, and the fracture was fixed by inserting a sterilized wire into the medullary cavity. Micro‐computed tomography (µCT) was performed at every week and samples were taken 4 weeks post‐fracture for histological analysis.

### Distraction Osteogenesis Model

The mouse model of DO of the femur was modified according to the previously reported protocols.^[^ ^60^ ^]^ In brief, after being anaesthetized with isoflurane, all animals were subjected to a right femur transverse osteotomy procedure at the mid‐shaft near the fibula‐tibia junction under sterile condition. An external fixator (RISYSTEM AG, SWITZERLAND) was applied to fix proximal and distal segments of the osteotomy site. An osteotomy was also made in the right fibula. Surgical incisions were then sutured sequentially. All mice were randomized into two groups with 6 in each group. All groups had bone lengthening started at day 7 postoperatively with 0.25 mm increments every 12 h, twice a day for seven days, for a total lengthening distance of 3.50 mm. Animal wellbeing and condition of surgical site were checked every three days. Samples were taken 6 weeks post‐surgery for histological analysis.

### Oral Administration

The mice were given orally 100 mg kg^−1^ YC‐SRT or YC‐RSV 1 day post fracture operation, once every 2 days, for three consecutive times. Four weeks post‐fracture, euthanize the mice for histological analysis. The other group injected with the same amount of PBS or YC were used as the control.

### Intra‐Callus Injection

The mice were given 500 ul GelDa or RSV@GelDa during fracture surgery. 4 weeks post‐fracture, euthanize the mice for histological analysis. The other group injected with the same amount of resveratrol free hydrogel (GelDa) were used as the control. The callus region injection of Yoda1 or the same amount of solvent were performed as previously described,^[^ ^34^ ^]^ 50 µg mL^−1^, 2 µL/time, once every two days, for 5 consecutive times, all the injections were given on the 4 days post fracture or 7 days post DO surgery.

### µCT Analysis

Samples were harvested at different time points post‐fracture or distraction, and fixed in 4% paraformaldehyde (PFA). Micro‐CT analyses were performed using a high‐resolution µCT scanner (Bruker SkyScan 1176, Karlsruhe, Germany). The 3D reconstruction of the fracture callus was performed using CT‐Vox 2.1. Three‐dimensional morphological parameters were recorded and calculated using CTAN 1.12 software (Bruker).^[^ ^61^ ^]^

### Histological Staining

Femur samples were fixed in 4% PFA at 4 °C for 24 h, decalcified with 10% EDTA (pH 7.4) for 3 weeks at 4 °C and paraffin‐embedded and 5‐µm thick sagittal sections were cut. Hematoxylin‐eosin (H/E), Safranin O‐Fast green (SO/FG) and TRAP staining was performed as previously described.^[^ ^62^, ^63^, ^64^ ^]^

### Immunohistochemistry and Immunofluorescence and Confocal Analyses

Specimens were prepared as previously described.^[^ ^65^, ^66^ ^]^ For immunohistochemistry (IHC), After antigen‐retrieved, BSA blocking and overnight incubation of the primary antibody, and incubated with the secondary antibody coupled with horseradish peroxidase (HRP), stained with DAB (Abcam), and restained with hematoxylin. Finally, images were taken under a microscope. For immunofluorescence (IF), After antigen‐retrieved overnight, permeabilized with 0.2% Triton X‐100, blocked with 2% BSA blocking and overnight incubation of the primary antibody, and incubated with anti‐rabbit Alexa Fluor 488 (Invitrogen) or anti‐mouse Alexa Fluor 568 (Invitrogen) secondary antibodies for 1 h at room temperature. The fluorescence signal was determined using a confocal microscope (Leica SP8 Confocal Microscopy System). Quantitative analysis of IHC and IF staining was performed in a double‐blind manner.

### Cell Viability Assay

Before the intervention of all compounds in cells, cell activity tests were conducted to screen for safe concentrations for use. The detection method was as previously described.^[^ ^67^ ^]^

### Cell Transfection

Small interfering RNA (siRNA) oligonucleotides and untargeted interfering control siRNA for mouse Piezo1 were purchased from GenePharma. The cell transfection method is as described above.^[^ ^68^ ^]^ The oligonucleotide sequence of Piezo1 siRNA is: For: GCUUGCUAGAACUUCACGUTT; Rev: ACGUGAAGUUCUAGCAAGCTT.

HEK293T cells were seeded in a six‐cell plate, and after 60% fusion, 2.5 µg plasmid was transfected with lipo3000 (Thermo Fisher Scientific, Inc.) in Opti MEM (Thermo Fisher Scientific, Inc.). Eight hours after transfection, culture with fresh culture medium and then continue for 48 h before corresponding treatment.

### Construct Mutation Piezo1 Plasmids

The mouse Piezo1 plasmid was purchased from MIAOLING BIOLOGY. Site‐directed mutagenesis of human and mouse Piezo1 was undertaken using a custom protocol with the high‐fidelity polymerase.

### Fluid‐Induced Flow Shear Stress Treatment

Fluid‐induced flow shear stress (FSS) was performed with Streamer System STR‐4000 (Flexcell International Corporation, Burlington, NC, USA) as previously described.^[^ ^69^ ^]^ For FSS treatment group, the culture slips were transferred into a parallel plat flow chamber and cells were exposed to 5 dyne cm^−2^ fluid flow for 2 h. For static controls, ATDC5 were kept in incubator without any further treatment. Cell and protein samples were collected after FSS treatment.

### Co‐Immunoprecipitation Assay

Co‐immunoprecipitation (Co‐IP) assay was performed as previously described.^[^ ^45^ ^]^ Briefly, Cells were transfected with corresponding expression plasmids. After 24 h, the cells were subjected to IP lysis buffer (Thermo Fisher) containing protease inhibitor cocktail and TSA/NAM to extract protein supernatant, and then incubated overnight with the corresponding primary antibody and Protein A/G magnetic beads at 4 °C. The Dynabheads‐antigen‐antibody complex was collected using Magnetic Stand (Thermo Fisher). The complex was washed with IP buffer for three times, re‐suspended with 1×loading buffer, boiled at 95 °C for 5 min, and perform Western blotting.

### Western Blot Analysis

Western blot analysis was performed as previously described.^[^ ^70^ ^]^ Extract cellular proteins, sequentially undergo electrophoresis, membrane transfer, incubation with primary and secondary antibodies, and detect protein expression with enhanced chemiluminescence kit (ECL kit, Bio‐Rad, USA).

### Calcium Imaging

Cell fluorescence was measured under excitation light of 340 and 380 nm as described.^[^ ^34^ ^]^ The fold change of [Ca^2+^]i was expressed as the 340/380 ratio before and after treatment. Cells with a [Ca^2+^]i fold change greater than 1.05 were defined as responsive cells.

### Structural Prediction and Visualization Comparison of Protein–Protein Complexes

AlphaFold was capable of predicting the combined structures of complexes, including proteins, nucleic acids, small molecules, ions, and modified residues, triggering a revolution in the modeling of protein structures and their interactions.^[^ ^71^ ^]^ It first used AlphaFold 2 to predict the structure of Piezo1 under different intervention conditions, then employed ChimeraX 1.9 for protein structure visualization to analyze the positions and lengths of hydrogen bonds. Additionally, Chimera 1.18 was used to perform structural superimposition and sequence alignment of different Piezo1 conformations to analyze the potential molecular mechanism by which Sirt1 deacetylation activates Piezo1.^[^ ^72^ ^]^

### Statistical Analyses

All experiments were performed at least three times. All data were presented as mean ± standard deviation and analyzed or plotted using GraphPad Prism 8.0. Differences between the two groups were analyzed using Student's *t*‐test. For comparisons between more than two groups, one‐way analysis of variance (ANOVA) and Tukey's or Dunnett's multiple comparison test were used. The value of *P* < 0.05 was considered statistically significant.

## Conflict of Interest

The authors declare no conflict of interest.

## Author Contributions

D.G., Y.R., H.P., Q.Y., W.Z., and B.Z. contributed equally to this work. G.X. and D.G. performed Study design. D.G., Y.R., H.P., Q.Y., W.Z., B.Z., P.X., R.L., H.X., W.J., T.H., Q.J. and L.Q. performed Study conduct and data collection. D.G., Y.R., H.P., Q.Y., W.Z., B.Z., R.L., F.Y.L., D.C. and C.L. performed Data analysis. G.X. and D.G. performed Data interpretation. G.X. and D.G. performed Drafting the manuscript. Y.R., H.P., Q.Y., W.Z. and B.Z. take the responsibility for the integrity of the data analyses.

## Supporting information



Supporting Information

Supporting Information

## Data Availability

The data that support the findings of this study are available from the corresponding author upon reasonable request.
