# Supplementary material for: The Sirt1‐Piezo1 Axis Promotes Bone Formation and Repair in Mice
Source: Adv Sci (Weinh). 2025 Sep 26;12(44):e10103. doi: 10.1002/advs.202510103 (PMC12667516; doi:10.1002/advs.202510103)
Supplement: Supplementary file 1 — Supporting Information [file ADVS-12-e10103-s002.docx]

**Supplementary Information**

**Supplementary Figure Legends**

**Supplementary Fig. 1. Piezo1 is upregulated in chondrocytes and linked to osteogenesis during fracture repair.** (A) RNA velocity of chondrocyte lineage. (B, C) Expression levels of Aggrecan, Piezo1 and Piezo2 in MSC, osteogenic lineage cell (OLC), and chondrocyte. (D, E) Expression levels of Piezo1 and Piezo2. (F) UMAP visualization of stromal cells at single cell level. (G) Feature plots showing marker gene expression used for cell type annotation. We used Ihh, Hpgd, Col10a1, Mmp13, Acan, Sox9, Col9a1, and Col2a1 as chondrogenic markers; Bglap, Alpl, Sp7, Runx2, Postn, and Col1a1 as osteogenic markers; and Cxcl12, Pdgfrb, and Pdgfra as mesenchymal stem cell (MSC) markers to assist in cell type identification. (H) Proportion of stromal cells at post fracture day 14 and control. (I) Functional enrichment analysis of chondrocytes related to osteogenesis. (J-N) Differential expressions of Osx, Col1a1, Alp, Vegfa and Piezo1 in chondrocytes from control and post fracture day 14. (O) Representative images of SO/FG and IHC staining of PIEZO1 in human cartilage calluses or cortical bone. Scale bar: 0.5 cm. (P) Quantification of PIEZO1-positive cells in calluses or cortical bone based on (O). MSC, mesenchymal stem cell; OLC, Osteolineage cell.

**Supplementary Fig. 2.** **Piezo1 loss in chondrocytes inhibits the endochondral ossification of the growth plate**. (A-C) Representative images of μCT, SO/FG and TRAP staining of growth plate in Con and cKO mice at 1 and 2 months post TAM injection. Scale bar: 200 μm. (D) The growth plate thickness (GP Th.) quantified of 1 and 2 months post TAM injection mice. (E) The thicknesses (Th.) of the proliferative zone (PZ) and hypertrophic zone (HZ) in the growth plate of 1 month post TAM injection mice. (F) Quantification of the TRAP staining by osteoclast surface per bone surface (Oc.S/BS) of 1 month post TAM injection mice. (G) Quantification of the TRAP staining by Oc.S/BS of 2 months post TAM injection mice. (H) Representative 3D reconstruction images of mice knee joint in Con and cKO group at 0/1/3 months post TAM injection (initial age: 2 month). Scale bar: 1.0 mm. (I) Representative images of IF staining of Col2a1, Runx2 and Osx in growth plate of each group mice at 2 months post TAM injection, scale bar: 200 μm. (J, K) Quantification of Runx2 and Osx positive cells in growth plate based on the IF staining results in (I). N = 6 per group. Results are expressed as mean ± standard deviation (s.d.). ***P* < 0.01.

**Supplementary Fig. 3. Structural modeling and comparison of the Piezo1–Sirt1 protein complex.** (A) The structural diagram of the Piezo1-Sirt1 protein complex predicted by AlphaFold 2 and visualized using ChimeraX 1.9. Sirt is displayed in light red, Piezo1 in dark blue. The amino acid residues of Piezo1 interacting with Sirt1 are shown in black, and the hydrogen bonds between Sirt1 and Piezo1 are represented by red dashed lines, with the corresponding hydrogen bond distances shown in red. (B) Perform a multiple sequence alignment to analyze the conservation of these lysine residues across homologous Piezo1 species. (C) Structural superimposition of Piezo1 variants with lysine mutations or Sirt1 modulation was performed using Chimera version 1.18. In each panel, the protein names shown above and below the dividing line represent the two structures being compared. For example, in the first column, the upper label denotes Piezo1 alone, while the lower label indicates Piezo1 bound to Sirt1, corresponding to the comparison "Piezo1 vs. Piezo1+Sirt1." The 3D structures of the two Piezo1 proteins are depicted in cyan and tan, respectively. WT refers to wild-type Piezo1, and S1 indicates Sirt1. K, Q, and R represent lysine, glutamine, and arginine residues, respectively. Irregular loops or enclosed regions represent the Sirt1 structure, the Sirt1 labels in red or green font represent Sirt1 from each of the two protein structures being compared, respectively. The central pore is labeled in white text.

**Supplementary Fig. 4.** **Comparison of spatial changes and distance matrices of different Piezo1 conformations.** (A–I) Spatial superimposition and structure-based sequence alignment of the central pore region of Piezo1 under different conformations. In each panel, the upper and lower sequences correspond to the samples indicated in the panel title. For example, in the first panel, the upper sequence aligns with the central pore region of Piezo1 in the "Piezo1 vs. Piezo1+Sirt1" comparison, while the lower sequence corresponds to the Piezo1 central pore in the Sirt1-bound structure. (J–R) Residue–residue distance maps of Piezo1 under various conformational and intervention conditions. P1 denotes Piezo1, and S1 denotes Sirt1. K, Q, and R represent lysine, glutamine, and arginine residues, respectively.

**Supplementary Fig. 5. Reciprocal regulation of Sirt1 and Piezo1 expression.** (A) Western blot of the effects of different concentrations of Yoda1 on Sirt1 protein expression in ATDC5 cells.

**Supplementary Fig. 6. Preparation and characterization of yeast-based drug delivery systems YC-SRT and YC-RSV.** (A) Schematic diagram showing the fabrication of yeast microcapsules (YCs), cationic nanoparticles (NPs) and YC-SRT. (B) SEM image of YC-SRT. Scale bar: 1 mm. (C) TEM image of YC-SRT. Scale bar, 1 mm. (D) SEM image of PEI-SRT. Scale bar, 1.5 mm. (E) Zeta potential distribution of YC-SRT. (F) Size distribution by intensity of YC-SRT. (G) Fluorescence image characterization of the Janus coating of Cy5.5 (red) on the YC-SRT. Scale bar: 2.5 mm. (H) The encapsulation efficiency and loading content of SRT (means±SD; N = 5). (I) In vitro drug release profiles of YC-SRT. Cumulative release of SRT from YC-SRT at pH 6.6 (simulating the acidic microenvironment of the fracture site) and pH 7.4 (simulating the normal bone microenvironment). (J) Schematic diagram showing the fabrication of yeast microcapsules (YCs), cationic nanoparticles (NPs) and YC-RSV. (K) SEM image of YC-RSV. Scale bar: 1 mm. (L) TEM image of YC-RSV. Scale bar: 2 mm. (M) SEM image of PEI-RSV. Scale bar: 1.5 mm. (N) Zeta potential distribution of YC-RSV. (O) Size distribution by intensity of YC-RSV. (P) Autofluorescence image characterization of the YC-RSV. Scale bar: 2.5 mm. (Q) The encapsulation efficiency and loading content of RSV (means±SD; N = 5). (R) In vitro drug release profiles of YC-RSV. Cumulative release of SRT from YC-RSV at pH 6.6 (simulating the acidic microenvironment of the fracture site) and pH 7.4 (simulating the normal bone microenvironment). N = 3 per group. Results are expressed as mean ± standard deviation (s.d.).

**Supplementary Fig. 7. Toxicity determination of potion-carrying YC-SRT/RSV in vivo.** (A) The histological appearance of the heart, liver, spleen, lung and kidney of mice after 4 weeks of local treatment with Con, YC-SRT and YC-RSV. (B) The ratio of visceral mass to body weight of mice in the three groups. N = 6 per group. Results are expressed as mean ± standard deviation (s.d.). ***P* < 0.01. (C) Representative images of H/E staining of the heart, liver, spleen, lung and kidney sections of the three groups mice. scale bar: 200 μm.

**Supplementary Fig. 8. Toxicity determination of potion-carrying gel in vivo and in vitro.** (A) The histological appearance of the heart, liver, spleen, lung and kidney of mice after 4 weeks of local treatment with Con, GelDa and RSV@GelDa. (B) the ratio of visceral mass to body weight of mice in the three groups. N = 6 per group. Results are expressed as mean ± standard deviation (s.d.). ***P* < 0.01. (C) Representative images of H/E staining of the heart, liver, spleen, lung and kidney sections of the three groups mice. scale bar: 200 μm. (D) CCK8 assay was used to detect the effects of different concentrations of RSV@GelDa sustained release solution on ATDC5 activity. Every group represents the average response of 3-dish repeats. Error bars are shown in colors.

**Supplementary Table 1:** Reagent information

| **Antibody** | **Company** | **catalog** | **Dilution** |
| --- | --- | --- | --- |
| Piezo1  Piezo1  Sirt1  Ac-K  Flag  His  Gapdh  Col1a1  Osx  Runx2  Opn  Vegfa  Kindlin-2  Talin  Vinculin  Yoda1  Resveratrol  SRT2104  3-TYP  Panobinostat | NOVUS  Proteintech  Proteintech  CST  Proteintech  Proteintech  ZSGB-BIO  Proteintech  Abcam  CST  Abcam  Proteintech  Proteintech  Proteintech  Proteintech  Medchemexpress  Medchemexpress  Medchemexpress  Medchemexpress  Medchemexpress | NBP1-78446  15939-1-AP  60303-1-Ig  9441  20543-1-AP  66005-1-Ig  TA-08  66761-1-Ig  ab209484  12556S  ab183910  19003-1-AP  11453-1-AP  14168-1-AP  66305-1-Ig  HY-18723  HY-16561  HY-15262  HY-108331  HY-10224 | IF (1:200), IHC (1:200)  WB (1:1000)  WB (1:1000) , IP (1:200)  WB (1:1000)  WB (1:1000), IF (1:200)  WB (1:1000), IF (1:200)  WB (1:1000), IF (1:200)  IF (1:200)  WB (1:1000)  WB (1:1000)  WB (1:1000)  WB (1:1000)  WB (1:1000)  WB (1:1000)  WB (1:1000)  1mM  5mM  5mM  1mM  10nm |

**Supplementary Table 2:** Human tissue information

| **Gender** | **Age** | **Fracture site** | **Time (week)** |
| --- | --- | --- | --- |
| Female | 34 | Ankle joint | 2 |
| Female | 66 | Femoral neck | 6 |
| Female | 46 | Tibia | 2 |
| Male | 28 | Humeral head | 3 |
| Female | 55 | Distal radius | 5 |
| Male | 55 | Ankle joint | 4 |

**Abbreviations**

Mesenchymal stem cell (MSC)

Osteolineage cell (OLC)

Distraction osteogenesis (DO)

SRT2104 (SRT)

Resveratrol (RSV)

Conditional knockout (cKO)

Silent information regulator 1 (SIRT1)

Specific-pathogen-free (SPF)

Tamoxifen (TAM)

Gelatin-Dopamine (Gelda)

Resveratrol @Gelatin-Dopamine hydrogel（RSV@Gelda hydrogel）

High Performance Liquid Chromatography (HPLC)

Micro-computed tomography (mCT)

Bone mineral density (BMD)

Total volume (TV)

Bone volume (BV)

Bone volume/tissue volume (BV/TV)

Trabecular thickness (Tb.Th)

Trabecular separation (Tb.Sp)

Bone surface/bone volume (BS/BV)

Hematoxylin-eosin(H/E)

Safranin O-Fast Green (SO/FG)

Immunofluorescence (IF)

Immunohistochemistry (IHC)

Region of Interest (ROI)

Small interfering RNA (siRNA)

Flow shear stress (FSS)

Horseradish peroxidase (HRP)

Small interfering RNA (siRNA)

Analysis of Variance (ANOVA)

Wild type (WT)

Week (W)

Osteopontin (OPN)

Runt-related transcription factor 2（RUNX2）

Osterix (OSX)

Cortical bone (Ct.B)

Cartilage (Cg)

Callus (Ca)

Muscle (Mu)

vehicle (Veh)

Woven bone (Wo.B)

Bone marrow (B.M)

Osteoclast surface per bone surface (Oc.S/BS)

Growth plate (GP)

Yeast microcapsules (YCs)

Nanoparticles (NPs)
