# Supplementary figures and images for: The Sirt1‐Piezo1 Axis Promotes Bone Formation and Repair in Mice
Source: Adv Sci (Weinh). 2025 Sep 26;12(44):e10103. doi: 10.1002/advs.202510103 (PMC12667516; doi:10.1002/advs.202510103)

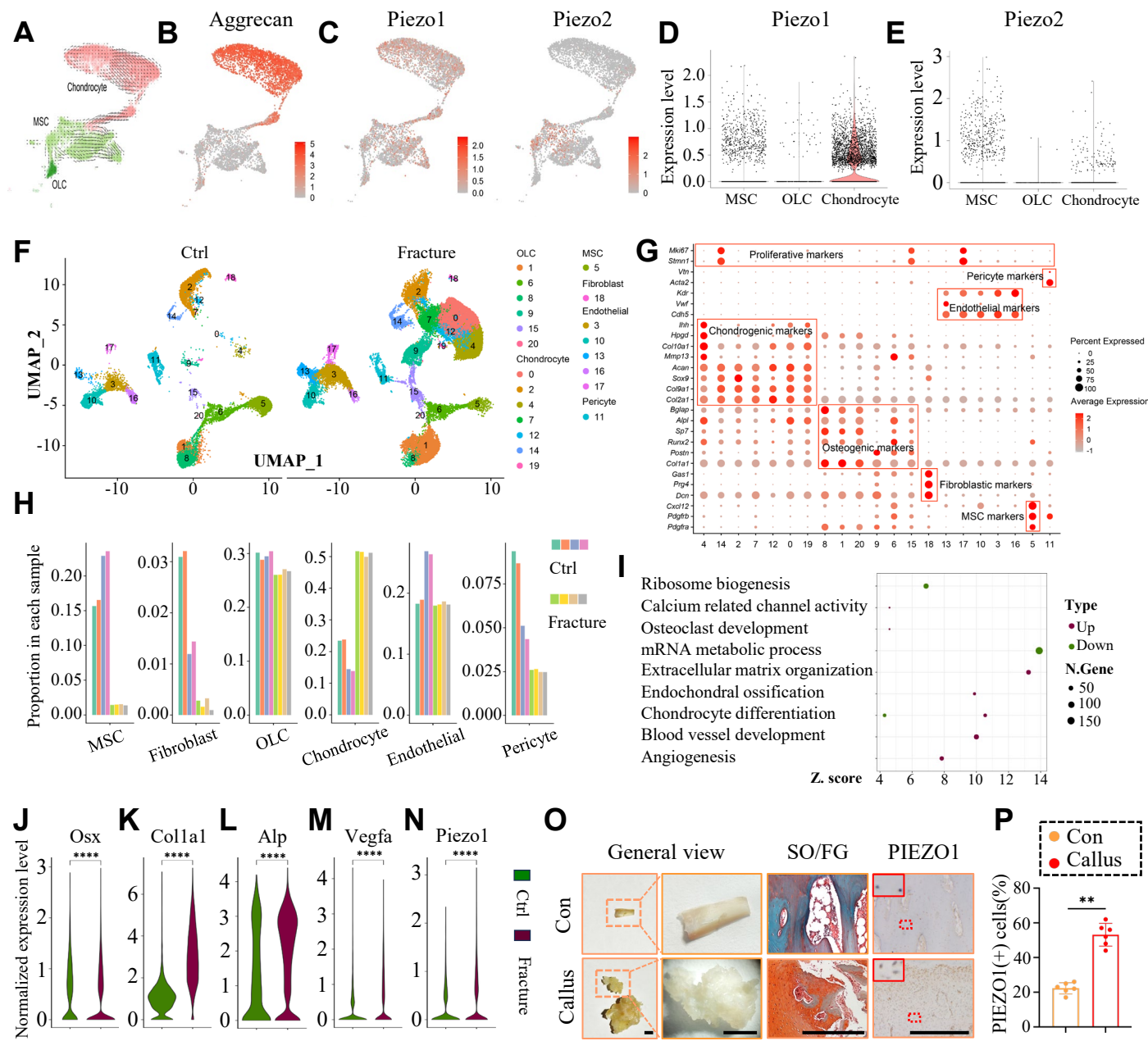

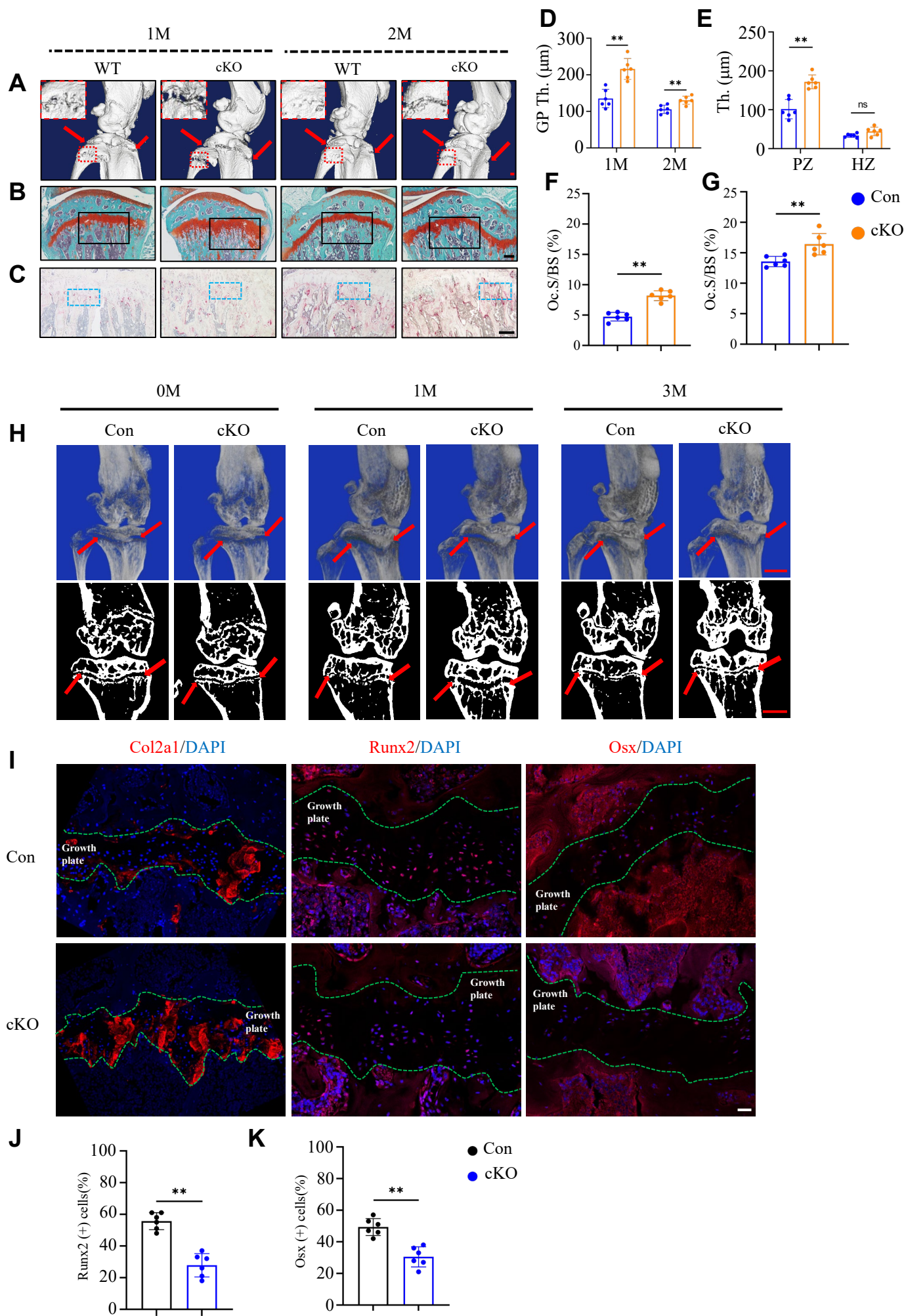



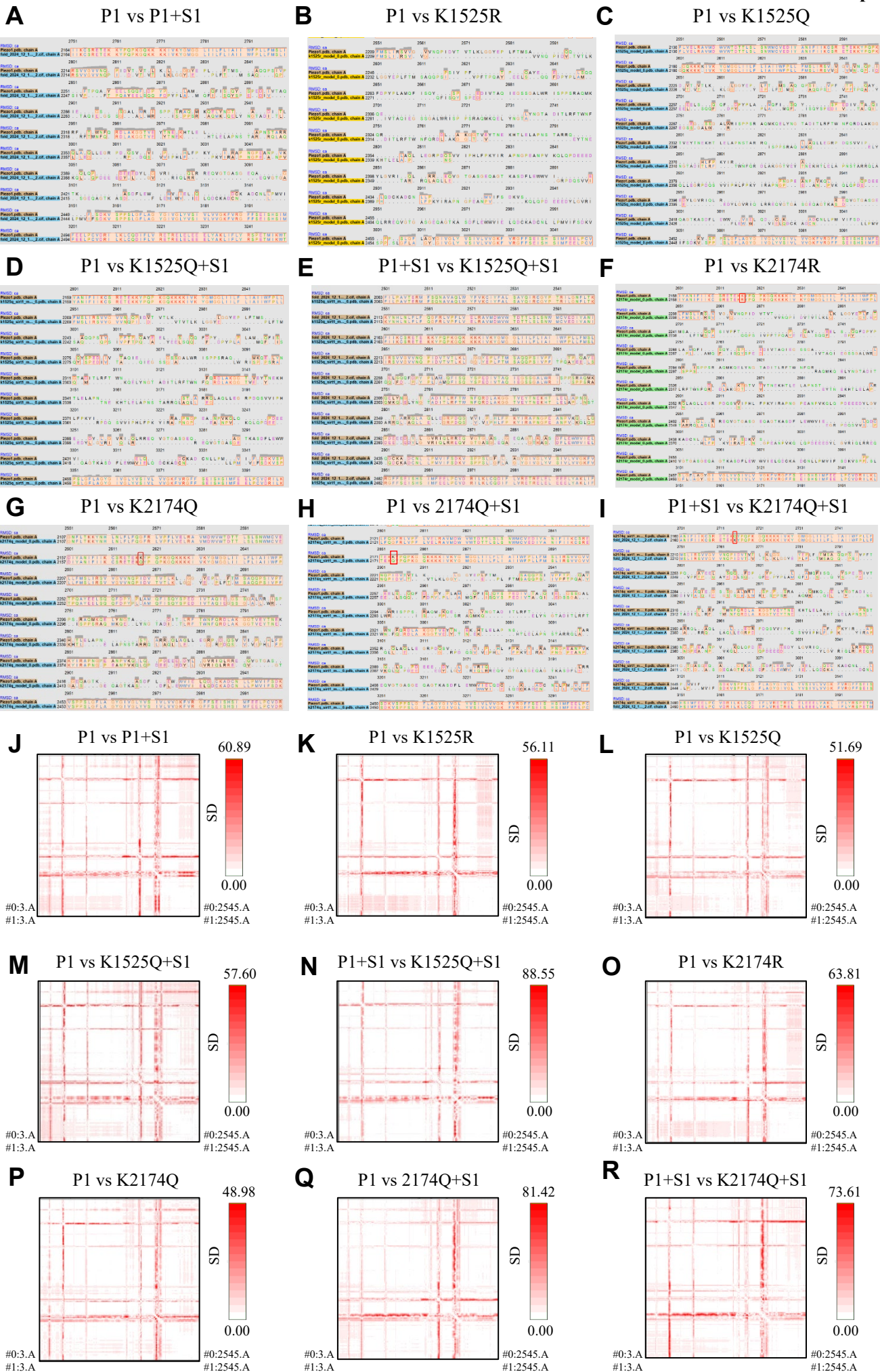

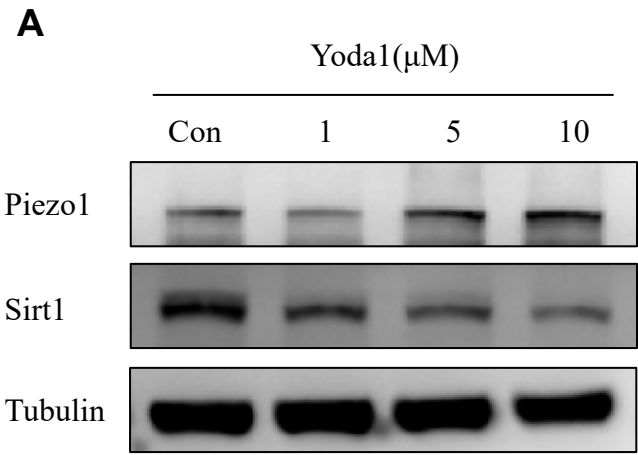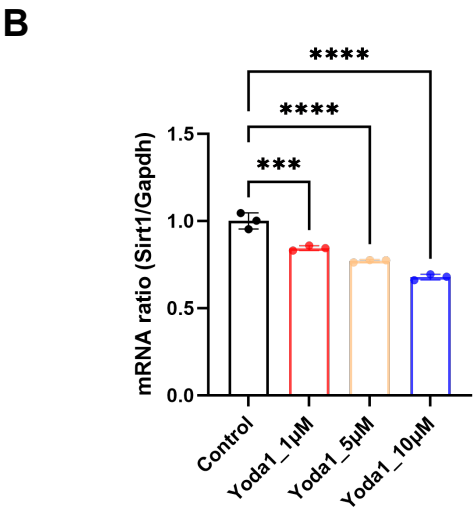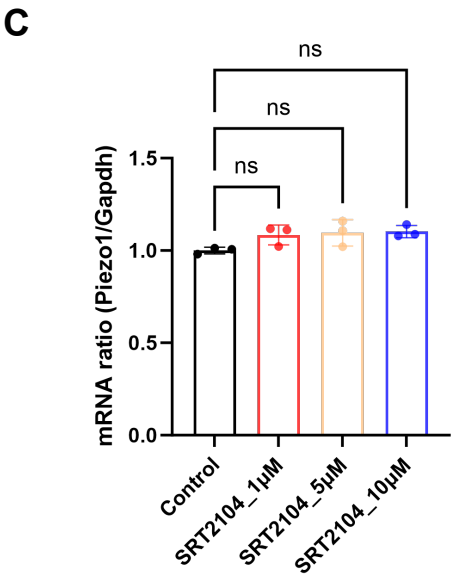

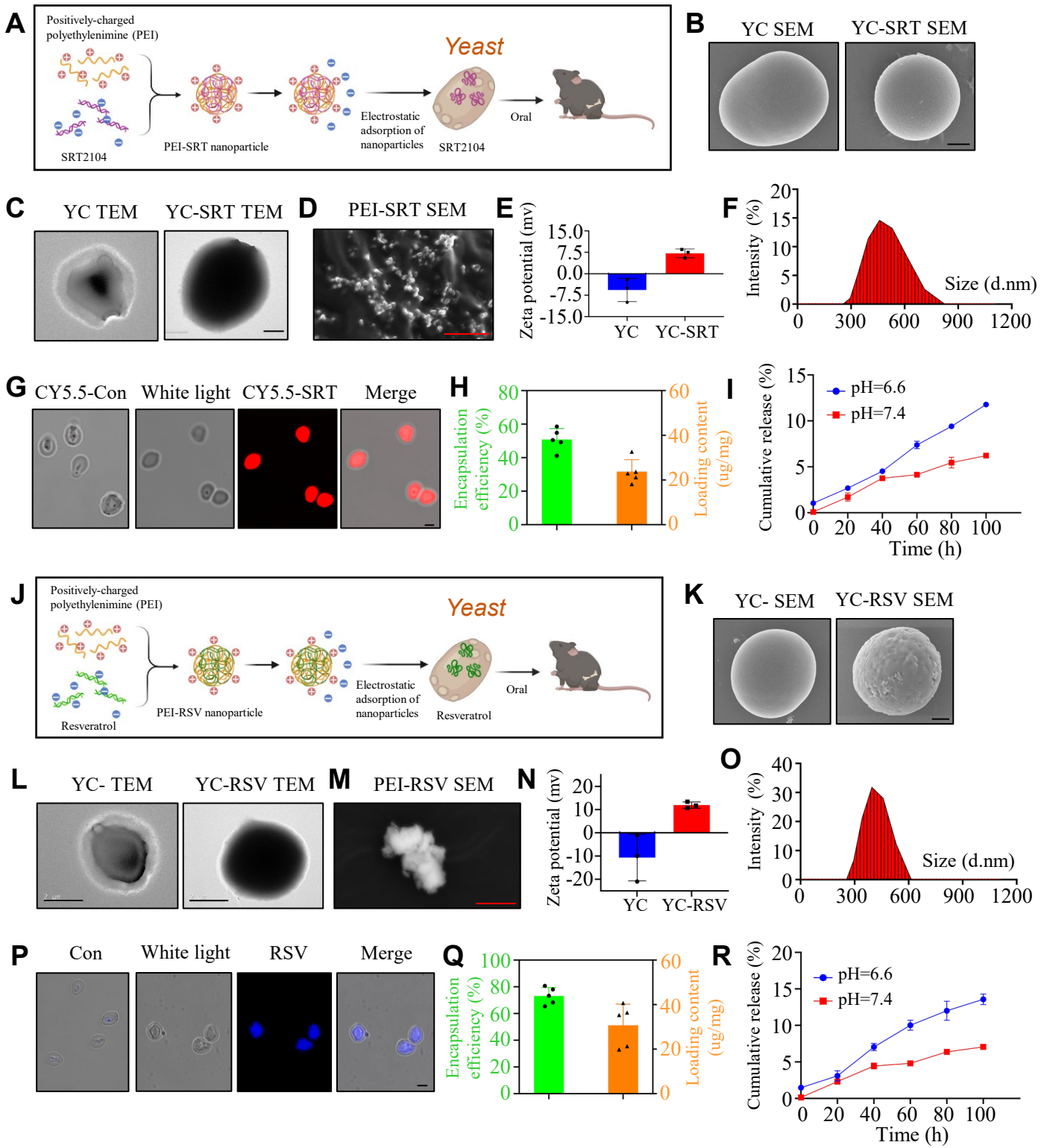

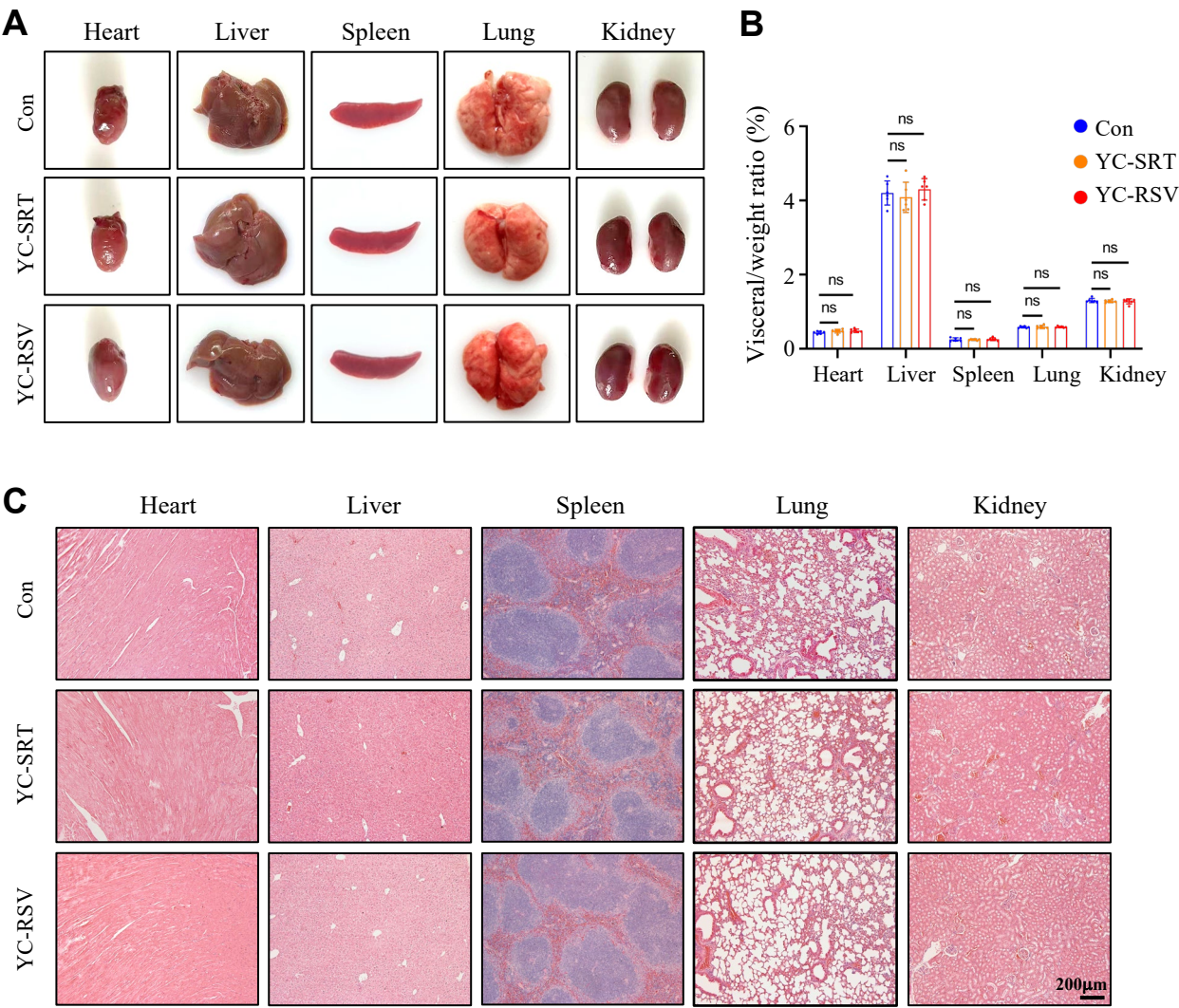

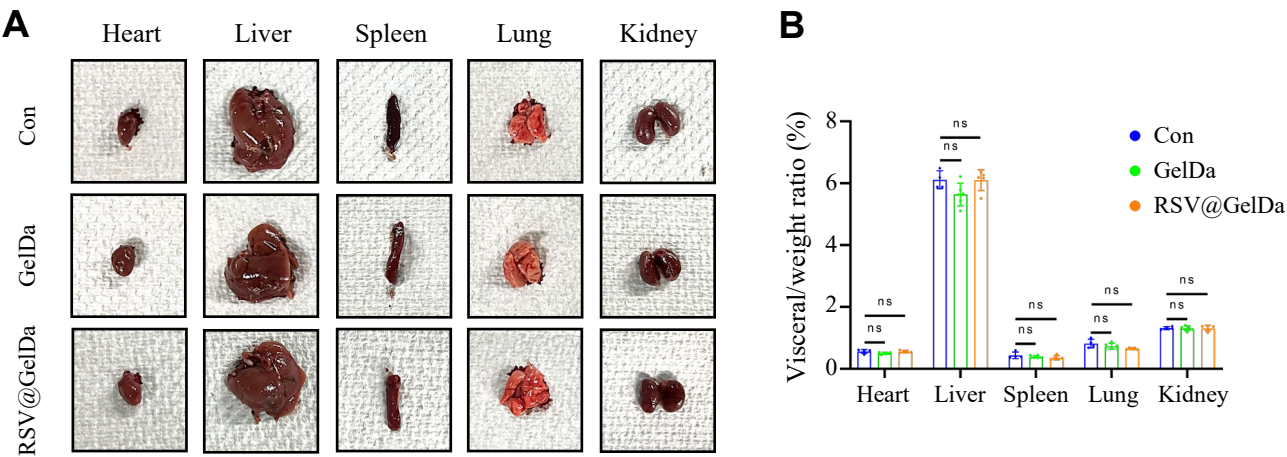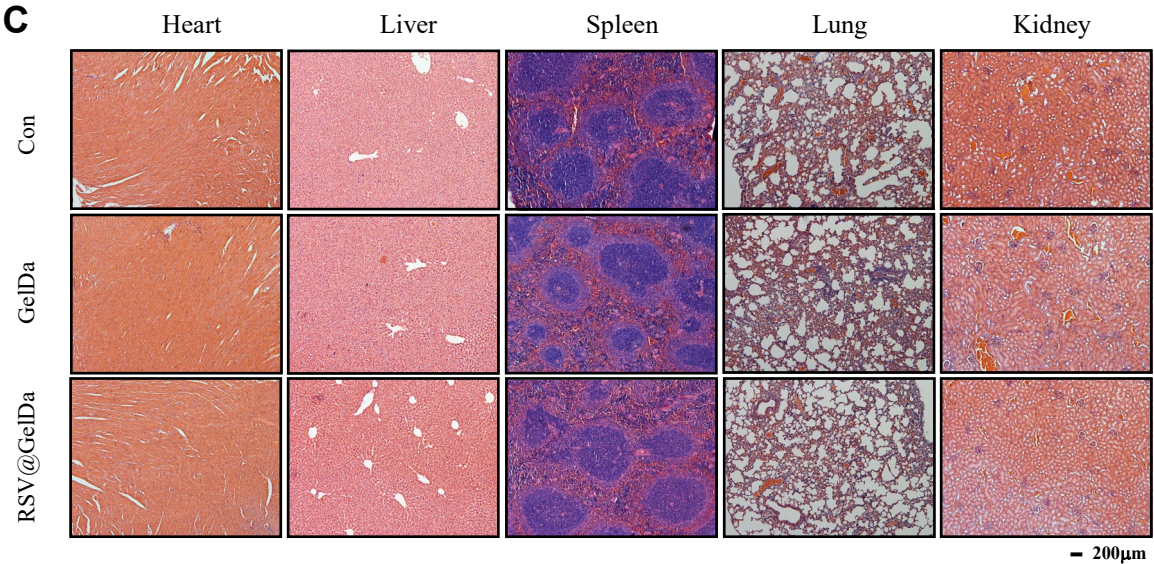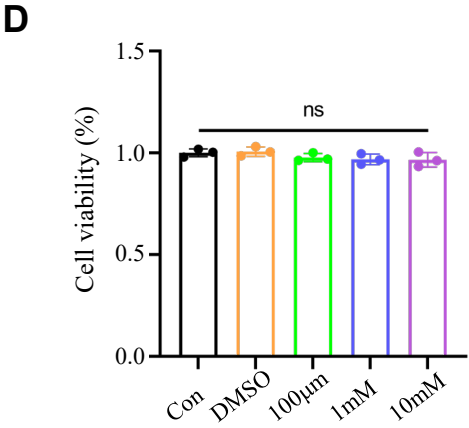

Supplement: Supplementary file 2 — Supporting Information [file ADVS-12-e10103-s001.pdf]
